# Expression and Differentiation between OCT4A and Its Pseudogenes in Human ESCs and Differentiated Adult Somatic Cells

**DOI:** 10.1371/journal.pone.0089546

**Published:** 2014-02-24

**Authors:** Mojca Jez, Sakthikumar Ambady, Olga Kashpur, Alexandra Grella, Christopher Malcuit, Lucy Vilner, Primoz Rozman, Tanja Dominko

**Affiliations:** 1 Blood Transfusion Centre of Slovenia, Ljubljana, Slovenia; 2 Department of Biomedical Engineering, Worcester Polytechnic Institute, Worcester, Massachusetts, United States of America; 3 Department of Biology and Biotechnology, Worcester Polytechnic Institute, Worcester, Massachusetts, United States of America; 4 Bioengineering Institute, Worcester Polytechnic Institute, Worcester, Massachusetts, United States of America; 5 CellThera, Inc., Worcester, Massachusetts, United States of America; Baylor College of Medicine, United States of America

## Abstract

The POU5F1 gene codes for the OCT4 transcription factor, which is one of the key regulators of pluripotency. Its transcription, alternative splicing, and alternative translation leading to the synthesis of the active, nuclear localized OCT4A has been described in detail. Much less, however, is known about actively transcribed OCT4 pseudogenes, several of which display high homology to OCT4A and can be expressed and translated into proteins. Using RT-PCR followed by pseudogene-specific restriction digestion, cloning, and sequencing we discriminate between OCT4A and transcripts for pseudogenes 1, 3 and 4. We show that expression of OCT4 and its pseudogenes follows a developmentally-regulated pattern in differentiating hESCs, indicating a tight regulatory relationship between them. We further demonstrate that differentiated human cells from a variety of tissues express exclusively pseudogenes. Expression of OCT4A can, however be triggered in adult differentiated cells by oxygen and FGF2-dependent mechanisms.

## Introduction

Octamer binding protein OCT4 (POU5F1), a POU family transcription factor transcribed from *POU5F1* gene is critical for maintenance of totipotency in primate blastomeres [*1*], [*2*] and for pluripotency of the inner cell mass of developing mammalian embryos [*1*], [3]–[*6*]. This critical role of OCT4 is maintained in embryonic stem cells [*7*]–[*10*], the developing germline [*11*]–[*13*], and germline tumors, such as seminomas and embryonic teratocarcinomas [*14*]–[*18*]. When inducing pluripotency in differentiated cells, OCT4 appears to be an essential, but not the only required transcription factor [*19*], [*20*]. OCT4 acts as a transcriptional activator of genes involved in maintenance of undifferentiated state and as a repressor of differentiation-specific genes [*7*], [21], [*22*].

In addition to its presence in embryonic and germ-derived cells, expression of OCT4 and other pluripotency markers has been reported in cancers, adult stem cells and various adult somatic tissues. The most abundant reports about OCT4 expression can be found for hematopoietic stem cells and mesenchymal stem cells from umbilical cord blood and bone marrow [*23*]–[*28*]. Detection of OCT4 transcripts in these compartments has led to a suggestion that embryonic OCT4A may participate in maintenance of stemness in adult stem cell compartments [*29*], somatic tissue cancers (bladder [*30*], squamous cell carcinoma [*31*], breast carcinoma [*32*], [*33*] and may even be expressed in normal differentiated tissues [*34*], [*35*]. Most astoundingly, existence of true pluripotent embryonic stem-like cells in adult organs has been proposed [*23*]–[25], [36], [*37*]. We have described expression of OCT4 in adult human fibroblasts undergoing induction into a regeneration competent cell type (iRC) using low oxygen culture and medium supplementation with FGF2 [*35*], [38], [*39*]. iRC cells display increased life span, maintain normal karyotype, express a number of stem cell genes, participate in in vivo regeneration of skeletal muscle, but do not form tumors [*39*]. While we observed that the amount of OCT4 transcript does not change before and after induction, we at the time did not examine whether the transcript belonged to the OCT4A or its pseudogenes.

**Figure 1:**
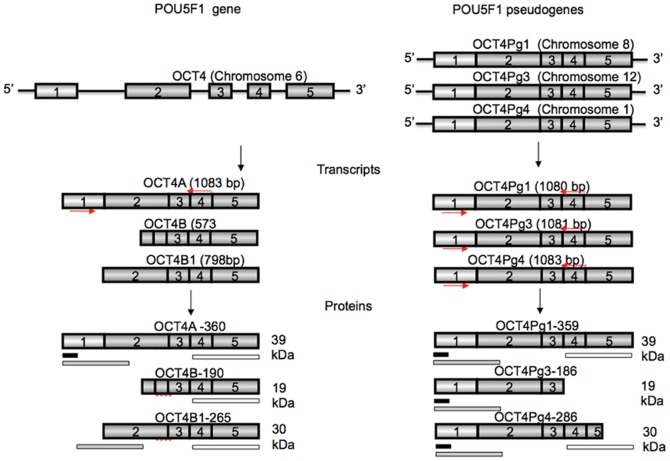
Schematic representation of OCT4 and its pseudogenes, transcript variants, and proteins. Red arrows indicate sequence against which the OCT4 primers were designed. Black rectangle – antigen peptide for sc5279 antibody; Grey rectangle - antigen peptide for ab19857 antibody; White rectangle - antigen peptide for ab18976 antibody.

The *POU5F1* gene produces three transcripts by alternative splicing [*40*], [*41*] and three protein isoforms by alternative codon use [*42*] (Figure 1). Only the OCT4A transcript contains the first exon at its 5′ sequence and only protein translated from this transcript consequently contains the unique N-terminal transactivation domain that regulates DNA binding of OCT4A to target promoters. It is only this full length OCT4A - a 360 amino acid protein - that localizes to the nucleus, binds to OCT4-regulated promoters, and contributes to the maintenance of pluripotency and self-renewal [*43*]. OCT4A transcript and protein can be distinguished from the non-pluripotency variants (OCT4B) using primers specific for 5′ sequence of OCT4A mRNA and N-terminal-recognizing antibodies, respectively, as well as by their distinct subcellular localization [*41*], [42], [44], [*45*].

With a more recent description of OCT4 pseudogenes and their expression [*46*]–[*48*], however, studies examining OCT4 expression have to consider an additional level of complexity (Table S1, Figure S1). Eight OCT4 pseudogenes have been identified using bioinformatics approach [*47*] and several of these are expressed and translated into proteins in somatic tumors, but not in embryonic carcinomas, fibroblasts or normal tissues [*48*]–[*51*]. Among the OCT4 pseudogenes, OCT4-pg1, OCT4-pg3 and OCT4-pg4 pseudogenes share a high sequence homology to OCT4A and share the unique N-terminal coding sequence. Similarly, cell types isolated from umbilical cord or adult bone marrow express OCT4-pg3, OCT4-pg4, and OCT4-pg5 but do not express embryonic OCT4A [*52*], and reports identifying these transcripts as OCT4A may have been erroneous. The only adult, non-cancer cell type that has been reported to express pseudogenes are cells derived from peripheral blood [*36*]. It has been suggested that pseudogenes may play regulatory roles in expression of genes from which they originate, potentially by an RNA interference-like mechanisms [*53*], [*54*] or even as weak transcriptional activators [*48*].

When studying expression of OCT4 it is therefore critically important to account for OCT4 transcripts, OCT4 pseudogene transcripts and all the possible protein products, the majority of which have no demonstrated DNA-binding activity. While methods and reagents to distinguish between OCT4A and OCT4B transcripts and protein variants are available, unavailability of similar reagents to distinguish between embryonic OCT4A and its pseudogene transcripts and proteins remains an obstacle to studying activity of these genes in more detail. Here we describe unique endonuclease restriction sites within OCT4A and its pseudogenes that enable differentiation between their transcripts, and investigate their expression in undifferentiated and differentiated human cells.

We demonstrate that embryonic stem cells express exclusively OCT4A and that during differentiation this expression ceases and is replaced by expression of pseudogenes. Similar regulation, however faster, takes place in teratocarcinoma cells. We also show that all differentiated cell types express exclusively pseudogenes, but this expression can shift to predominantly embryonic OCT4 in adult human fibroblasts undergoing induction of regenerative competence (iRC) by FGF2 and low oxygen.

## Materials and Methods

### Cell Culture

Human embryonic stem cells (hESCs, WA09) and induced pluripotent stem cells (iPSCs, DF19-9-7T) were obtained, cultured, and maintained as recommended by the supplier (WiCell). For differentiation into embryonic bodies (EBs), stem cells were either trypsinized (hESCs) or mechanically dissociated (iPSCs) and plated onto low attachment culture plates in differentiation medium consisting of DMEM-F12, 7.5% serum replacement, 10% FBS, 0.5× NEAA (Gibco), 0.005% β-mercaptoethanol (Gibco), and 2 µmol of retinoic acid (Sigma). Medium was changed with fresh medium twice a week. On day 5 of differentiation, EBs were transferred to cell culture plates where they were cultured until day 21. NTERA, NCCIT (both teratocarcinoma), HeLa, and NHSY (glioblastoma) cells were obtained from the American Tissue Culture Collection (ATCC). For differentiation, NTERA cells were seeded at density 1×10^6^ cells per 75 cm^2^ in expansion medium (DMEM +10% FBS). After attachment the medium was changed to differentiation medium consisting of DMEM +10% FBS supplemented with 0.01 mM trans-retinoic acid (Sigma). Medium was changed twice a week and samples for RNA isolation taken during 21 days post-differentiation.

Other cell types or their total RNA was obtained from Lonza (human mesenchymal stem cells – hMSCs, human umbilical cord vascular endothelium - HUVEC), Clontech (testis RNA), Lonza (smooth muscle cells, and Clontech (RNA from heart). All cell types were cultures as recommended by suppliers. Control adult primary human fibroblasts CRL-2352 (ATCC) were cultured in DMEM-F12, 10% FBS at ambient oxygen, or in 2% oxygen in the same medium, supplemented with 4 ng/mL of FGF2 (PeproTech).

### Total RNA Extraction and RT-PCR

Total RNA was extracted using TRIZOL reagent (Invitrogen). RNA integrity was examined on 1% agarose gel. 1 µg of total RNA was reverse transcribed using QuantiTech Reverse Transcription kit (Qiagen) according to the manufacturer’s protocol. Total RNA was first treated with DNA Wipeout buffer to remove potential contamination of genomic DNA and then used for reverse transcription. The cDNA samples were stored at −20°C. The polymerase chain reaction contained 2× GoTaq Green Mastermix (Promega), 20 pmols of primers and PCR water. The PCR primers used for PCR were: NANOG F: 5′-TGTCTTCTGCTGAGATGCCTCACA-3′ and R: 5′-CCTTCTGCGTCACACCATTGCTAT-3′; CRIPTO1 F: 5′-GACGAGCAAATTCCTGATGG-3′ and R: 5′-GCCTCTTTTCCCCCTAATTG-3′; GAPDH F: 5′-ATCACCATCTTCCAGGAGCGA-3′ and R: 5′-TTCTCCATGGTGGTGAAGACG-3′; OCT4 F, 5′- GTTGATCCTCGGACCTGGCTA-3′; R, 5′- GGTTGCCTCTCACTCGGTTCT-3′. The OCT4 primers were designed to bind at nucleotides 142–162 and 768–788 of the coding OCT4 sequence and amplify OCT4 as well as pseudogene 1, 3 and 4 transcripts (Figure 1). PCR cycling parameters consisted of an initial cDNA denaturation of 2 min at 95°C, followed by 15 sec at 94°C, 30 sec at annealing temperature 60°C, and DNA extension for 1 min at 72°C for 31 cycles. A final extension step was performed at 72°C for 10 min and held at 4°C until analysis. All PCR reactions included no template controls or no RT reaction. PCR products were resolved by 1% agarose gel electrophoresis and were visualized by exposure to 535 nm light on Kodak MM4000 image analyzer.

### PCR Clean-up

Four 25 microliter reactions were combined and PCR product cleaned using NucleoSpin Extract II kit (Macherey-Nagel). Briefly, PCR reactions were diluted with 2 volumes of Buffer NT and loaded on NucleoSpin Extract II column and centrifuged 1 min at 11,000×g. Flow-through was discarded and column washed with 700 µL of buffer NT3 and then the membrane was dried for 2 min at 11,000×g. DNA was eluted into a new 1.5 mL tube and DNA concentration was determined with Nanodrop.

### Restriction Digest

**Figure 2:**
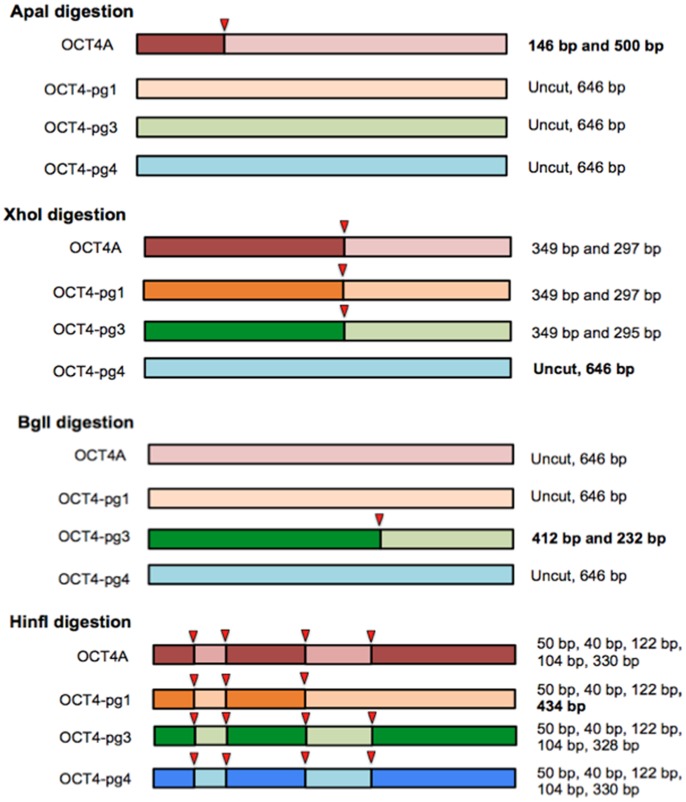
Schematic representation of restriction sites in 646-PCR amplicon. Specific restriction sites were used to distinguish between embryonic OCT4A transcript and different pseudogenes. Red arrows show restriction sites. **ApaI** restriction site is present only in embryonic OCT4A and can be used to distinguish embryonic form from all six pseudogenes; after restriction, a 146 bp and 500 bp long fragments are produced. **HinfI** digestion results in several smaller fragments among which the 434 bp fragment is specific only for OCT4-pg1. **BglI** digests only OCT4-pg3 into two fragments of 412 bp and 232 bp. **XhoI** does not digest OCT4-pg4.

Sequence alignments between OCT4A and OCT4-pg1, OCT4-pg3 and OCT4-pg4 were used to identify restriction sites that were then used for differentiation between the transcripts (Figure 2). A previously described ApaI restriction site specific for embryonic OCT4A [*55*] and three novel restriction sites for pseudogenes were selected (HinfI for OCT4-pg1; BglI for OCT4-pg3, XhoI for OCT4-pg4; all from NEBiolabs). 500 ng of PCR amplicons were digested with 12.5U ApaI, 10U XhoI, 10U BglI, or 10U HinfI. ApaI digestion reaction was incubated for 1.5 h at room temperature and the other three for 1.5 h at 37°C. Digested products were separated on 2% agarose gel with ethidium bromide and visualized by exposure to 535 nm light on a Kodak MM4000 image analyzer.

### Cloning and Sequencing

PCR amplicons were cloned into pCR4-TOPO vector (TOPO TA Cloning Kit for Sequencing, Invitrogen), were transformed into TOPO10 competent *E. coli*, and were then plated onto LB-agar plates containing 100 µg/ml ampicillin. Antibiotic resistant colonies were selected, expanded, and plasmid was isolated, and bi-directionally sequenced from both T3 and T7 promoters (Genewiz). Three clones were sequenced for OCT4A from ESCs, 3 clones for each pseudogene from control fibroblasts and 6 clones for OCT4A from treated fibroblasts.

### Western Blotting

Total cell lysates were prepared using common procedures. Nuclear and cytoplasmic fractions were prepared as follows: cells were collected using 0.05% trypsin (Cellgro) and washed twice with PBS and pelleted. Buffer A (50 mM NaCl, 10 mM HEPES pH8.0, 500 mM sucrose, 1 mM EDTA, 0.5 mM spermidine, 0.15 mM spermine, 0.2% Triton X-100, 7 mM 2-mercaptoethanol) supplemented with complete protease inhibitor cocktail (Roche) was used to isolate cytoplasmic fractions. The cell pellets were then washed with buffer B (50 mM NaCl, 10 mM HEPES pH8.0, 25% glycerol, 0.1 mM EDTA, 0.5 mM spermidine, 0.15 mM spermine, 7 mM 2-mercaptoethanol) supplemented with complete protease inhibitor cocktail (Roche). Buffer C (350 mM NaCl, 10 mM HEPES pH8.0, 25% glycerol, 0.1 mM EDTA, 0.5 mM spermidine, 0.15 mM spermine, 7 mM 2-mercaptoethanol) supplemented with complete protease inhibitor cocktail (Roche) was used to isolate nuclear extracts. Protein concentration was quantified using Bradford Protein Assay Kit (Thermo Scientific). Lysates, nuclear and cytoplasmic fractions were separated on 10% SDS-PAGE, and transferred onto PVDF membrane using Towbin’s transfer buffer. Membranes were blocked with 5% dry milk and 5% FBS in TTBS and incubated with primary antibodies diluted in blocking buffer. Primary anti-human OCT4 antibodies used were rabbit polyclonal ab19857 derived against the C-terminus of human OCT4, rabbit polyclonal ab18976 derived against amino acids 1–14 (both from Abcam); and mouse monoclonal sc-5279 (Santa Cruz Biotechnology) developed against the N-terminus. The anti-α-tubulin antibody (Sigma) was used as a loading control. Appropriate secondary HRP-conjugated antibodies were used and signal detected by enhanced chemilluminescence (Amersham, BioRad) on Kodak MM4000.

### Immunocytochemistry

The same three anti-OCT4 antibodies were used for ICC: sc-5279 (Santa Cruz Biotechnology), ab19857, and ab18976 (both from Abcam). The following secondary antibodies were used: anti-mouse AlexaFluor568 and anti-rabbit AlexaFluor568. AlexaFluor488-conjugated phalloidin (Invitrogen) was used to stain actin filaments and DAPI to stain DNA. Images were obtained with appropriate excitation/emission on Leica confocal microscope using identical image acquisition settings.

## Results

### The Pool of OCT4 Transcripts in Various Cell Types is Composed of Messages Originating from its Gene and its Pseudogenes

**Figure 3:**
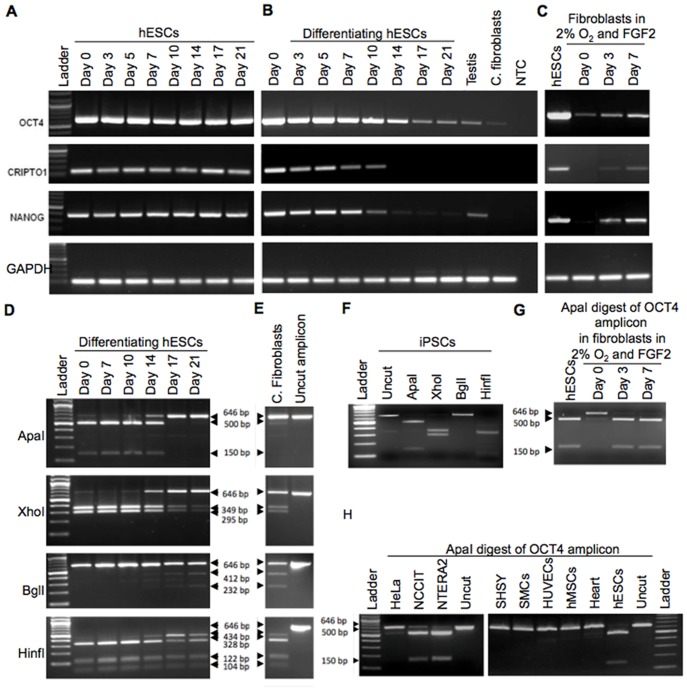
RT-PCR for expression of embryonic stem cell specific genes. OCT4, NANOG and CRIPTO1 in human ESCs during culture (A), during 21 days after induction of hESC differentiation (B), and in adult human dermal fibroblasts cultured in 2% oxygen with FGF2 supplementation (C). Restriction digest of 646 bp OCT4 amplicon from human embryonic stem cells (D), control fibroblasts (E), and iPSCs (F). ApaI restriction digest of OCT4 amplicon in fibroblasts grown in 2% oxygen and FGF2 supplementation (G), and various transformed, multipotent and differentiated cells (H). NCCIT – teratocarcinoma, NTERA2– teratocarcinoma, SHSY – neuroblastoma, SMCs – smooth muscle cells, HUVECs – human umbilical vein endothelial cells, hMSCs – human mesenchymal stem cells. Only the embryonic OCT4A contains ApaI restriction site.

To establish a baseline expression levels for stem cell genes in hESCs and iPSCs, we first examined expression of OCT4, NANOG and CRIPTO1 by RT-PCR. As expected, strong expression of all three stem cell genes was maintained during hESC (Figure 3A) and iPSC maintenance culture (data not shown). Starting 10 days after induction of differentiation, the expression of these embryonic markers gradually decreased and was undetectable for CRIPTO1 on day 14 and NANOG on day 17. Low levels of OCT4 transcript, however persisted and were still detectable on day 21 (Figure 3B). Expression of the same genes in control adult human fibroblasts resembled the expression of these genes in day 21 differentiated hESCs. We next examined whether expression of these genes can be detected in adult human fibroblasts cultured in 2% oxygen culture in the presence of FGF2. RT-PCR amplified transcripts could be detected for all three genes after 3 days and increased on day 7 (Figure 3C), confirming our previously published observations [*37*] *.*

**Figure 4:**
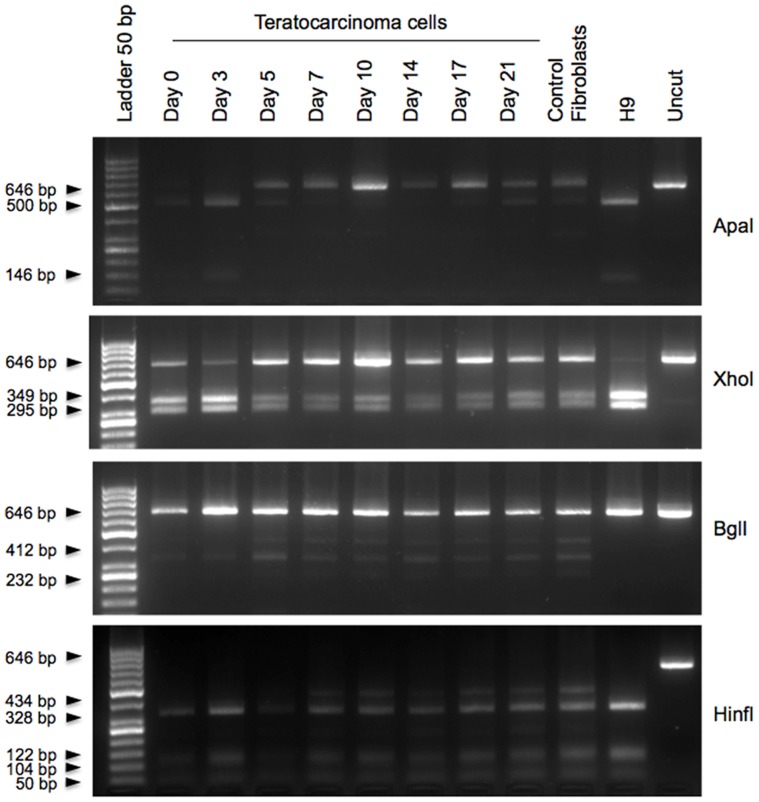
Restriction digest of 646 bp OCT4 amplicon from human embryonic carcinoma cells during 21 days of differentiation in medium containing retinoic acid.

In addition to recognizing the OCT4A, our OCT4 primers were designed to amplify a fragment including 5′ region which is common for OCT4A and pseudogenes 1, 3 and 4, but which is missing in all other pseudogene transcripts and OCT4B transcripts (Figure 1). The expected 646 bp amplicon, therefore could contain any of the four transcripts. This allowed us to examine the OCT4 amplicon in these cells in more detail. To identify the composition of the transcripts that could contribute to the 646 bp OCT4 amplicon at any time and to distinguish between the individual transcripts, we searched otherwise highly homologous sequences of OCT4 and OCT4-pg1, 3 and 4 for potentially unique endonuclease restriction sites. In addition to previously described ApaI site, that is specific only for OCT4A [*55*], we were able to identify three restriction sites that were then selected for differentiation between pseudogene transcripts (Figure 2). Restriction analysis of the amplicon from hESCs during differentiation is presented in Figure 3D. ApaI restriction analysis of the 646 bp PCR product identified the persistence of OCT4A until day 14 of differentiation, as evident by ApaI restriction of the amplicon into 146 and 500 bp fragments. By day 17 the amplicon could not be digested by ApaI any longer. This indicated that the embryonic OCT4A transcript was no longer present and the entire 646 bp amplicon on day 17 and after consisted of pseudogene transcripts. Digestion of the amplicon with XhoI, which digests all but the OCT4-pg4 indicated very low amounts of this transcript in hESCs and started gradually increasing between days 10 and 14 after differentiation. Digestion with BglI which cuts only OCT4-pg3 (412 and 232 bp fragments) revealed a gradual increase of this transcript 14 days after differentiation. Lastly, digestion with HinfI which cuts all transcripts into 5 fragments except OCT4-pg1 (4 fragments) similarly indicated the start of OCT4-pg1 transcription on day 14 by the virtue of the longer, 424 bp fragment unique to OCT4-pg1. Overall, the amount of all pseudogene transcripts increased with a concomitant decease in transcription of OCT4A over the 21 days of hESC differentiation. The pattern of OCT4A and pseudogenes expression in control fibroblasts resembled the pattern observed in differentiated hESCs and control fibroblasts, which expressed only OCT4 pseudogenes (Figure 3E). However, ApaI digestion of the OCT4 amplicon from fibroblasts grown in 2% oxygen and with 4 ng/ml FGF2– the conditions we have previously used to induce a phenotype switch to a regeneration competent cell [*38*] - identified embryonic OCT4A transcript at both day 3 and day 7 of culture in these conditions, indicating a reversal of expression from OCT4 pseudogenes to embryonic OCT4A (Figure 3G), resembling the pattern of ApaI digestion in hESC and iPSCs (Figure 3D and 3F). When a variety of other cell types were examined, only hESCs and teratocarcinoma cell lines (NTERA and NCCIT) expressed ApaI-sensitive OCT4A transcript (Figure 3H). Similar downregulation of OCT4A transcript was observed during differentiation of teratocarcinoma cells (Figure 4), however, simultaneous expression of pseudogenes started increasing much earlier during differentiation, and could be observed from day 5 onward.

To confirm our restriction digestion results, the 646 bp PCR amplicons from hESCs, 2%O_2_/FGF2 treated fibroblasts and control fibroblasts were cloned into TOPO vector and three clones from each sample for each pseudogene and six clones from 2%O_2_/FGF2 treated fibroblasts were sequenced. Sequencing confirmed our restriction data; hESCs and 2%O_2_/FGF2 treated fibroblasts expressed only embryonic OCT4A, while control fibroblasts expressed OCT4-pg1, pg3 and pg4 (Tables S2–S7).

### Translation and Localization of Protein Products of OCT4 Gene and its Pseudogenes

**Figure 5:**
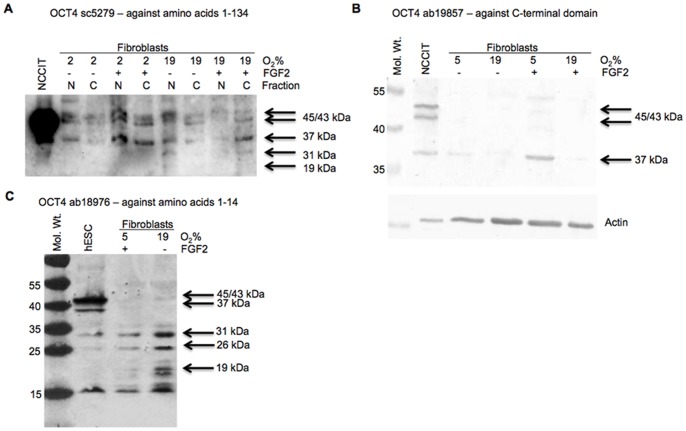
Protein expression of stem cell specific genes. Western blots for OCT4 in human embryonic stem cells (hESC), teratocarcinoma cells and adult human dermal fibroblasts (A, C) or muscle fibroblasts (B) using different antibodies. Complete cell lysates or nuclear and cytoplasmic fractions (N and C, respectively) were separated on 10% SDS PAGE, transferred to PVDF and blotted with different antibodies. Fibroblasts were grown for 7 days under low (2 or 5%) or ambient (19%) oxygen, in the presence or absence of FGF2 (+ and −, respectively).

To determine whether any of the OCT4 transcripts were, in fact, translated into detectable proteins, Western blotting was performed. As demonstrated in Figure 5A, the OCT4 sc-5279 antibody, directed against N-terminal protein sequence, recognized several bands in teratocarcinoma cells (NCCIT) and in fibroblasts, regardless of culture conditions. The doublet that migrated at 45/43 kDa (OCT4A or OCT4pg1) and a band migrating around 37 kDa (likely somatic OCT4B) were present in all samples, while the two faster migrating proteins (30 and 20 kDa) were present only in fibroblasts gown in ambient oxygen, regardless off FGF2 supplementation. The proteins were detected in both cytoplasmic and nuclear fractions of fibroblasts. Knowing that FGF2/2%O2 fibroblasts express OCT4A, however, it is reasonable to assume that some of the slower migrating doublet represents embryonic OCT4A. In control fibroblasts these bands are likely contributed by pseudogenes 1, 3 and 4, as the antibody should recognize sequences in the N-terminal of these proteins (Figure 5A). The second antibody, ab19857 (against C-terminal), similarly recognized the doublet of 45/43 kDa and a protein migrating around 37 kDa, but failed to recognize any other protein products. The amount of detected protein at 37 kDa was significantly higher in cells grown in low oxygen and potentiated by addition of FGF2 (Figure 5B). The third antibody ab18976, derived against the N-terminal OCT4 sequence could, in principle detect OCT4A and pseudogenes 1 and 3. Intensity of the band in treated fibroblast was significantly lower than in hESCs (Figure 5C). A 31 kDa band was detected in both control and treated fibroblasts and could represent OCT4pg4. The identity of the 26 kDa band detected with this antibody remains unknown. An additional 19 kDa protein, that could be the product pf OCT4pg3, was recognized in control fibroblasts, but was absent in stem cells and treated fibroblasts.

**Figure 6:**
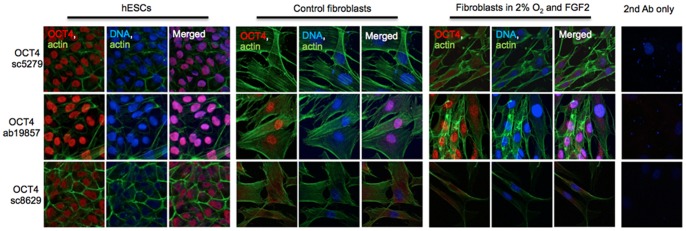
Immunocytochemistry of OCT4 in human embryonic stem cells (hESCs) and fibroblasts. Cells were cultured in control (ambient 19% O_2_, no FGF2) and 2% oxygen with FGF2 supplementation for 7 days. Cells were stained with three different OCT4 antibodies, as indicated. Red – OCT4, Green – actin; Blue – DNA.

Immunocytochemistry revealed OCT4 presence in cell nuclei of hESCs regardless of the antibody used, as expected (Figure 6). The N-terminal specific antibody sc-5279 recognized weak nuclear signal in 2%O_2_/FGF2 fibroblasts. The C-terminal ab19857 recognized nuclear localized protein in hESCs, and nuclear and cytoplasmic proteins in control and treated fibroblasts. As none of the OCT4B variants localize to the nucleus, the nuclear signal can be contributed only by OCT4A or pseudogene OCT4-pg1 derived protein [*48*], [*51*] *.* Considering the individual cell type transcript profile, it is possible that in treated fibroblasts the antibody recognized OCT4A. The OCT4-pg4 protein has yet to be detected by any anti-OCT4 antibody, and OCT4-pg3 localizes to the cytoplasm [*51*] *.* The sc-8629, another C-terminal specific antibody, recognized nuclear OCT4A in hESCs and cytoplasmic proteins in both control and treated fibroblasts. Cytoplasmic staining in both fibroblasts may be identifying OCT4A, OCT4B, OCT4pg1 [*42*] *,* and/or OCT4-pg3 [*51*] *.* These data corroborate our previously published results [*35*] and indicate that expression of OCT4 in somatic cells is under the control of an oxygen and FGF2-sensitive mechanism.

## Discussion

OCT4 has received considerable attention during the past years and its expression has been reported in various cell types and across a spectrum of differentiation cell states. Due to the possibility of multiple transcript variants and protein isoforms, as well as expressed pseudogenes being detected, more rigorous analyses of OCT4 are required [*44*], [*46*] *.* Existence of high number of pseudogenes for OCT4 is not an exception. Excessive frequency of pseudogenes for embryonic stem cells-specific genes was also described for Nanog (10 pseudogenes), Stella (16 pseudogenes), and Cripto-1 (6 pseudogenes) [*47*], [*52*] *.*

We identified expression of Oct-4pg1, Oct-4pg3 and Oct-4pg4 pseudogenes in normal adult human fibroblasts using PCR based approach followed by pseudogene-specific restriction digestion and sequencing. Our study confirms that expression of OCT4A protein is exclusive to hESCs and germ cell-derived tumor cell lines. None of the other examined somatic cells, somatic tumor cell lines or adult stem cells expressed OCT4A. In an exact opposite pattern, all cell types examined except hESCs and germ cell tumor-derived cell lines expressed pseudogenes 1, 3 and 4. Monitoring the relationship between OCT4A expression and expression of pseudogenes in hESCs during differentiation, an interesting pattern emerges; a gradual decrease of OCT4A occurs in a parallel with an increase in expression of pseudogenes. This may indicate a tight regulatory relationship between OCT4A and its pseudogenes, even though the OCT4 gene and all the pseudogenes are located on different chromosomes [*47*]. One of the pseudogenes, namely OCT4-pg3 is located within the 445 kb of NANOG, STELLA and GDF3 locus on chromosome 12 [*56*], significance of which still needs to be examined.

It is interesting that similar changes occur in reverse during acquisition of regeneration competence in adult human fibroblasts. After a short term culture in reduced oxygen and supplementation with FGF2 [*35*], [*38*] human fibroblast cease expressing OCT4 pseudogenes in favor of OCT4A. We have reported previously that these cells do not acquire the pluripotent phenotype [*35*], but rather undergo a phenotype switch that demonstrates regeneration competence *in vivo* [*38*]. Cells demonstrate an increased life span, doubled population doubling capacity, maintain a normal karyotype and do not form tumors in SCID mice. They do, however, demonstrate long term engraftment and acquire PAX7 positive phenotype when transplanted into a skeletal muscle defect in mice [*38*]. Significance and relevance of OCT4A expression, if any, during acquisition of regeneration competence, is not clear and is currently under investigation. Nevertheless, this is the first study in which a switch from pseudogene to OCT4A expression has been reported and induced by selective manipulation of culture conditions. We speculate that pseudogene silencing is induced in adult somatic cells using our culture system, an event that may be required for re-activation of OCT4A. While clearly not pluripotent, regeneration-competent cells express low levels of OCT4A. It has been shown that OCT4 is controlling pluripotency in a quantitative manner [*57*]. Specifically, high level of OCT4 expression drives ES cells to endoderm and mesoderm lineages, while stem cells with low level of OCT4 differentiate into trophoectoderm. Only a specific level of OCT4 can maintain stem cells in a pluripotent state. These observations suggest that OCT4 is different from many known transcription regulators that appear to function in an on-off manner. Consequently, low levels of embryonic OCT4A may not be functionally relevant for either induction or maintenance of pluripotency, but may be relevant for induction of this novel regeneration-competent cell phenotype. Our data indicate that chromatin plasticity may be an inherent feature of adult fibroblasts and that the manifestation of this plasticity may be influenced by the extracellular environment. Our data also support the notion that regeneration competence does not depend on cell pluripotency and may be regulated by a novel, OCT4A-dependent mechanism. OCT4 gene ablation in stem cell compartments of several somatic tissues, including intestinal epithelium, bone marrow (hematopoietic and mesenchymal lineages), hair follicle, brain and liver in mice revealed no abnormalities in homeostasis or regenerative capacity indicating that OCT4 is dispensable for both self-renewal and maintenance of somatic stem cells in adult mammal. Neither OCT4 nor its pseudogenes, for example, are required for multipotency of human stromal cells from different tissues [*49*]. There is no common regulatory network that has been shown to exist between the various somatic stem cell types and that can be compared to gene regulatory network that maintains pluripotency in ES cells with OCT4 as its cornerstone [*52*], [*58*]. It is more likely that developmental potency of tissue-specific stem cells is governed by intrinsic and extrinsic signals from the microenvironment in which they exist [*59*].

Several reports argue that detection of OCT4A in somatic cells is an artifact; possibly a consequence of DNA contamination and/or inability of currently available methods to distinguish between OCT4A and its pseudogenes. Misidentification of OCT4 in somatic tissues and cancer cell lines can also result from detection of pseudogene transcripts by RT-PCR and/or nuclear detection of pseudogene proteins by ICC. Most studies indeed do not exclude the possibilities of pseudogene expression [*16*], [44], [46], [*59*] and do not distinguish between OCT4 isoforms [*46*] *.* All OCT4 protein isoforms are identical in their C-terminal region and distinct in N-terminal region so the location of the epitope recognized by any anti-OCT4 antibody should be considered first [*42*], [*46*] and combined with a diligent analysis of the OCT4 transcript. Adding the possibility that OCT4 pseudogenes translate into proteins and that OCT4-pg1 can localize to the nucleus [*51*] and confer transcriptional activation of some reporter gene constructs [*48*] *,* diligent analysis should be employed. This may likely require a combination of experimental approaches, such as RT-PCR, restriction analysis for various pseudogenes, and protein analysis. Additional functional studies, such as chromatin immunoprecipitation, electromobility shift assay and/or transcription analysis of downstream genes may need to be included to reach a valid conclusion.

Besides accurately characterizing OCT4 expression, relevance of OCT4 expression should be considered for each chosen experimental system. While OCT4A expression may be critically important when studying pluripotency and self-renewal, examining expression of OCT4A in studies involving stem cells from adult compartments or in somatic cell cancers may be of little functional relevance [*59*], [*60*] *.*

## Supporting Information

Figure S1 **Differences between Oct4 pseudogenes based on the coding sequences.** N-terminal domains from all pseudogenes are similar to embryonic Oct4.
(TIF)

Table S1 **OCT4 and its pseudogenes.** Source: http://www.ncbi.nlm.nih.gov/sites/entrez.
(DOCX)

Table S2 **CLUSTAL 2.1 multiple sequence alignment of Oct4A, Oct4pg1, Oct4pg3 and Oct4pg4 sequences.** Oct4 primers: F, 5′- GTTGATCCTCGGACCTGGCTA-3′; R, 5′- GGTTGCCTCTCACTCGGTTCT-3′; (reverse complement: AGAACCGAGTGAGAGGCAACC). Amplicon length is 646 bp. Primer sequences are shown in yellow, **bold** is the sequence amplified with these primers. Purple are polymorphisms in primer annealing sites. Nucleotide polymorphisms exploited for enzyme specific digestion are marked in red and the whole enzyme recognition site in green.
(DOCX)

Table S3 **Alignment of the cloned 646 bp amplicon amplified from embryonic stem cells (H9) – clone 1, 8 and 9– aligned to Oct4A mRNA sequence from GenBank (NM_002701.4).**
(DOCX)

Table S4 **Alignment of the 646 bp amplicon amplified from fibroblasts grown in 2%O_2_ and FGF2 (CRL2352 treated) – colony 1, 3, 4, 5, 6 and 7– aligned to Oct4A mRNA sequence from GenBank (NM_002701.4).**
(DOCX)

Table S5 **Alignment of the 646 bp amplicon amplified from control fibroblasts (CRL2352 untreated) – colony 12, 14 and 28– aligned to Oct4pg1 mRNA sequence from GenBank (NR_002304.2).**
(DOCX)

Table S6 **Alignment of the 646 bp amplicon amplified from control fibroblasts (CRL2352 untreated) – colony 3, 7 and 11– aligned to Oct4pg3 mRNA sequence from GenBank (NR_036440.1).**
(DOCX)

Table S7 **Alignment of the 646 bp amplicon amplified from control fibroblasts (CRL2352 untreated) – colony 2 and 5– aligned to Oct4pg4 mRNA sequence from GenBank (NR_034180.1).**
(DOCX)
