# Correction: Expression and Differentiation between OCT4A and Its Pseudogenes in Human ESCs and Differentiated Adult Somatic Cells

**DOI:** 10.1371/journal.pone.0104296

**Published:** 2014-07-28

**Authors:** Mojca Jez, Sakthikumar Ambady, Olga Kashpur, Alexandra Grella, Christopher Malcuit, Lucy Vilner, Primoz Rozman, Tanja Dominko

Due to an error in the preparation of Figure 3, the GAPDH panel for hESCs in Figure 3A is incorrect and duplicates the GAPDH panel for differentiating hESCs in Figure 3B.

**Figure 3:**
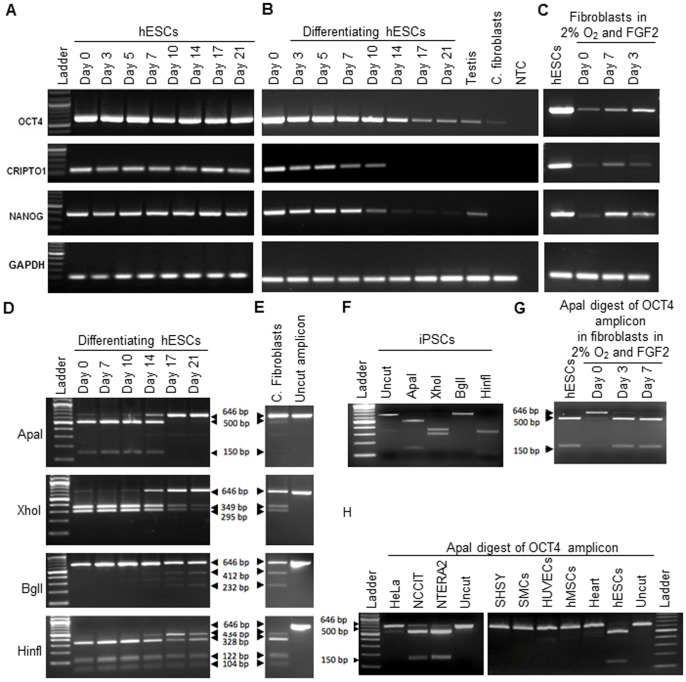
RT-PCR for expression of embryonic stem cell specific genes. OCT4, NANOG and CRIPTO1 in human ESCs during culture (A), during 21 days after induction of hESC differentiation (B), and in adult human dermal fibroblasts cultured in 2% oxygen with FGF2 supplementation (C). Restriction digest of 646 bp OCT4 amplicon from human embryonic stem cells (D), control fibroblasts (E), and iPSCs (F). ApaI restriction digest of OCT4 amplicon in fibroblasts grown in 2% oxygen and FGF2 supplementation (G), and various transformed, multipotent and differentiated cells (H). NCCIT – teratocarcinoma, NTERA2– teratocarcinoma, SHSY – neuroblastoma, SMCs – smooth muscle cells, HUVECs – human umbilical vein endothelial cells, hMSCs – human mesenchymal stem cells. Only the embryonic OCT4A contains ApaI restriction site.

The authors would also like to provide a clarification in relation to Figure 3C. The bands in the original gel for CRIPTO1 and NANOG were re-arranged to display them in the order d0, d3, d7 in the published figure, instead of the original loading order of d0, d7, d3.

The authors are supplying a corrected Figure 3 that includes the correct GAPDH panel for Figure 3A and a revised Figure 3C where the original order of the lanes is maintained. The authors apologize for the inaccuracies in the original Figure 3. These changes do not affect the results and conclusions reported in the article.
