# Supplementary figures and images for: Expression and Differentiation between OCT4A and Its Pseudogenes in Human ESCs and Differentiated Adult Somatic Cells
Source: PLoS One. 2014 Feb 24;9(2):e89546. doi: 10.1371/journal.pone.0089546 (PMC3933561; doi:10.1371/journal.pone.0089546)

**Table S1.** **OCT4 and its pseudogenes**

**
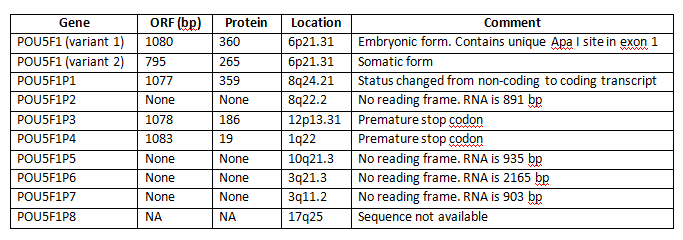
**

Source: http://www.ncbi.nlm.nih.gov/sites/entrez

Supplement: Table S1 — OCT4 and its pseudogenes. Source: http://www.ncbi.nlm.nih.gov/sites/entrez. (DOCX) [file pone.0089546.s002.docx]
