# Supplementary material for: Expression and Differentiation between OCT4A and Its Pseudogenes in Human ESCs and Differentiated Adult Somatic Cells
Source: PLoS One. 2014 Feb 24;9(2):e89546. doi: 10.1371/journal.pone.0089546 (PMC3933561; doi:10.1371/journal.pone.0089546)

**Table S2. CLUSTAL 2.1 multiple sequence alignment of Oct4A, Oct4pg1, Oct4pg3 and Oct4pg4 sequences**

gi|Oct4A|ref|NM_002701.4| --------------------------------------------------

gi|Oct4pg3|ref|NR_036440.1| --------------------------------------------------

gi|Oct4pg4|ref|NR_034180.1| --------------------------------------------------

gi|Oct4pg1|ref|NR_002304.2| AACATTTCCAAATCTTGGCATTCTTATCCACAAAGTGAAGATAATAATTG 50

gi|Oct4A|ref|NM_002701.4| --------------------------------------------------

gi|Oct4pg3|ref|NR_036440.1| --------------------------------------------------

gi|Oct4pg4|ref|NR_034180.1| --------------------------------------------------

gi|Oct4pg1|ref|NR_002304.2| TCAATTCACAGGTGATTATGATTTAAAGAGATTACTTTTGAAGAGTTCCT 100

gi|Oct4A|ref|NM_002701.4| --------------------------------------------------

gi|Oct4pg3|ref|NR_036440.1| --------------------------------------------------

gi|Oct4pg4|ref|NR_034180.1| --------------------------------------------------

gi|Oct4pg1|ref|NR_002304.2| AACACATTCAGTCAACATTTAATGATGCTTCAGGCACTGTGTTCATTGCT 150

gi|Oct4A|ref|NM_002701.4| --------------------------------------------------

gi|Oct4pg3|ref|NR_036440.1| --------------------------------------------------

gi|Oct4pg4|ref|NR_034180.1| --------------------------------------------------

gi|Oct4pg1|ref|NR_002304.2| AGTGAGCGTATGACACACACAGCCATACGGTCACAGAGCTTTCAATGAAA 200

gi|Oct4A|ref|NM_002701.4| -CCTTCGCAAGCCCTCATTTCACCAGGCCCCCGGCTTGGGGCGCCTTCCT 49

gi|Oct4pg3|ref|NR_036440.1| --------------------------------------------------

gi|Oct4pg4|ref|NR_034180.1| --------------------------------------------------

gi|Oct4pg1|ref|NR_002304.2| AGTAACATAATTGCTCATTTCACCAGGCCCCCGGCTTGGGGCGCCTTCCT 250

gi|Oct4A|ref|NM_002701.4| TCCCCATGGCGGGACACCTGGCTTCGGATTTCG-CCTTCTCGCCCCCTCC 98

gi|Oct4pg3|ref|NR_036440.1| ----CATGGCGGGACACCTGGCTTCGGATTTCG-CCTTCTCACCCCCTCC 45

gi|Oct4pg4|ref|NR_034180.1| -----ATGGCGGGACACCTGGCTTCGGATGCCTGCCTTCTTGCCCCCTCC 45

gi|Oct4pg1|ref|NR_002304.2| TCCCCATGGCGGGACACCTGGCTTCGGATTTCG-CCTTCTCGCCCCCTCC 299

************************ * ****** .********

gi|Oct4A|ref|NM_002701.4| AGGTGGTGGAGGTGATGGGCCAGGGGGGCCGGAGCCGGGCTGG**GTTGATC** 148

gi|Oct4pg3|ref|NR_036440.1| AGGCGGTGGAGGTGATGGGCCAGGGGGGCCGGAGCCGGGCTGG**GTTGATC** 95

gi|Oct4pg4|ref|NR_034180.1| AGGCGGTGGAGGTGATGGGCCAGGGGGGCCGGAGCCGGGCTGG**GTTGATC** 95

gi|Oct4pg1|ref|NR_002304.2| AGGCGGTGGGGGTGATGGGCCATGGGGGGCGGAGCCGGGCTGG**GTTGATC** 349

*** *****.************ ***** *********************

gi|Oct4A|ref|NM_002701.4| **CTCGGACCTGGCTAAGCTTCCAAGGCCCTCCTGGAGGGCCAGGAATCGGG** 198 **(HinfI)**

gi|Oct4pg3|ref|NR_036440.1| **CTCGGACCTGGCTAAGCTTCCAAGGCCCTCCTGGAGGGCCAGGAATCGGG** 145

gi|Oct4pg4|ref|NR_034180.1| **CTCGGACCTGGCTAAGCTTCCAAGGCCCTCCTGGAGGGCCAGGAATCGGG** 145

gi|Oct4pg1|ref|NR_002304.2| **CTCTGACCTGGCTAAGCTTCCAAGGCCCTCCTGGAGGGCCAGGAATCGGG** 399

*** **********************************************

gi|Oct4A|ref|NM_002701.4| **CCGGGGGTTGGGCCAGGCTCTGAGGTGTGGGGGATTCCCCCATGCCCCCC** 248 **(HinfI)**

gi|Oct4pg3|ref|NR_036440.1| **CCGGGGTTTGGGCCAGGCTCTGAGGAGTGGGGGATTCCCCCATGTCCCCC** 195

gi|Oct4pg4|ref|NR_034180.1| **CCGGGAGTTGGGTCAGGCTCTGAGGTGTGGGGGATTCCCCCATGCCCCCC** 195

gi|Oct4pg1|ref|NR_002304.2| **CCGGGGGTTGGGCCAGGCTCTGAGGTGTGGGGGATTCCCCCTTGCCCCCC** 449

*****. ***** ************:***************:** *****

gi|Oct4A|ref|NM_002701.4| **GCCGTATGAGTTCTGTGGGGGGATGGCGTACTGTGGGCCCCAGGTTGGAG** 298 **(ApaI)**

gi|Oct4pg3|ref|NR_036440.1| **GCCGTATGAGTTCTGCGGGGGGATGGCGTACTGTGGGCCTCAGACTGGAG** 245

gi|Oct4pg4|ref|NR_034180.1| **GCTGTATGAGTTCTGTGGGGGGATGGCGTACTGTGGGCCTCAGGTTGGAG** 245

gi|Oct4pg1|ref|NR_002304.2| **GCCGTATGAGTTATGTGGGGGGATGGCGTACTGTGGGCCTCAGGTTGGAG** 499

** *********.** *********************** ***. *****

gi|Oct4A|ref|NM_002701.4| **TGGGGCTAGTGCCCCAAGGCGGCTTGGAGACCTCTCAGCCTGAGGGCGAA** 348

gi|Oct4pg3|ref|NR_036440.1| **TGGGGCTAGTGCCCCAAGACGGCTTGGAGACCTCTCAGCCTGAGGGCGAA** 295

gi|Oct4pg4|ref|NR_034180.1| **TGCGGCTAGTGCCCCAAGGCGGCTTGGAGACCTCTCAGCCTGAGGGCGAA** 295

gi|Oct4pg1|ref|NR_002304.2| **TGGGGCTAGTGCCCCAAGGCGGCTTGGAGACCTCTCAGCCTGAGAGCGAA** 549

** ***************.*************************.*****

gi|Oct4A|ref|NM_002701.4| **GCAGGAGTCGGGGTGGAGAGCAACTCCGATGGGGCCTCCCCGGAGCCCTG** 398 **(HinfI)**

gi|Oct4pg3|ref|NR_036440.1| **GCAGGAGTCGGGGTGGAGAGCAACTCCGATGGGGCCTCCCCGGAGCCCTG** 345

gi|Oct4pg4|ref|NR_034180.1| **GCAGGAGTCAGGGTGGAGAGCAACTCCGATGGCACCTCCCTGGAGCCCTG** 345

gi|Oct4pg1|ref|NR_002304.2| **GCAGGAGTCGGGGTGGAGAGCAACTCCAATGGGGCCTCCCCGGAACCCTG** 599

*********.*****************.**** .****** ***.*****

gi|Oct4A|ref|NM_002701.4| **CACCGTCACCCCTGGTGCCGTGAAGCTGGAGAAGGAGAAGCTGGAGCAAA** 448

gi|Oct4pg3|ref|NR_036440.1| **CACCGTCCCCTCTGGTGCCGTGAAGCTGGAGAAGGAGAAGCTGGAGCAAA** 395

gi|Oct4pg4|ref|NR_034180.1| **CACCGTCCCCCCTGGTGCCGTGAAACTGGAGAAGGAGAAGCTGGAGCAAA** 395

gi|Oct4pg1|ref|NR_002304.2| **CACCGTCCCCCCTGGTGCCGTGAAGCTGGAGAAGGAGAAGCTAGAGCAAA** 649

*******.** *************.*****************.*******

gi|Oct4A|ref|NM_002701.4| **ACCCGGAGGAGTCCCAGGACATCAAAGCTCTGCAGAAAGAACTCGAGCAA** 498**(HinfI)(XhoI)**

gi|Oct4pg3|ref|NR_036440.1| **ACCCGGAGGAGTCCCAGGACATCAAAGCTCTGCAGAAAGAACTCGAGCAA** 445

gi|Oct4pg4|ref|NR_034180.1| **ACCCGCAGGAGTCCCAGAACATCAAAGCTCTGCAGAAAGAACTCGAACAA** 445

gi|Oct4pg1|ref|NR_002304.2| **ACCCGGAGAAGTCCCAGGACATCAAAGCTCTGCAGAAAGAACTCGAGCAA** 699

***** **.********.****************************.***

gi|Oct4A|ref|NM_002701.4| **TTTGCCAAGCTCCTGAAGCAGAAGAGGATCACCCTGGGATATACACAGGC** 548

gi|Oct4pg3|ref|NR_036440.1| **TTTGCCAAGCTCCTGAAGCAGAAGAGGATCACCCTGGGATATACACAGGC** 495 **(BglI)**

gi|Oct4pg4|ref|NR_034180.1| **TTTGCCAAGCTCCTGAAGCAGAAGAGGATCACCCTGGGATATACACAGGC** 495

gi|Oct4pg1|ref|NR_002304.2| **TTTGCCAAGCTCCTGAAGCAGAAGAGGATCACCCTGGGATATACACAGGC** 749

**************************************************

gi|Oct4A|ref|NM_002701.4| **CGATGTGGGGCTCACCCTGGGGGTTCTATTTGGGAAGGTATTCAGCCAAA** 598

gi|Oct4pg3|ref|NR_036440.1| **CGATGTGG--CTCACCCTGGGGGTTCTATTTGGGAAGGTGTTCAGCCAAA** 543

gi|Oct4pg4|ref|NR_034180.1| **CGATGTGGGGCTCACCCTGGGGGTTCTATTTGGGAAGGTGTTCAGCCAAA** 545

gi|Oct4pg1|ref|NR_002304.2| **CGATGTGGGGCTCATCCTGGGGGTTCTATTTGGGAAGGTGTTCAGCCAAA** 799

******** **** ************************.**********

gi|Oct4A|ref|NM_002701.4| **CGACCATCTGCCGCTTTGAGGCTCTGCAGCTTAGCTTCAAGAACATGTGT** 648

gi|Oct4pg3|ref|NR_036440.1| **CGACCATCTGCCGCTTTGAGGCTCTGCAGCTTAGCTTCAAGAACATGTGT** 593

gi|Oct4pg4|ref|NR_034180.1| **CGACCATCTGCCGCTTTGAGGGTCTGCAGCTTAGCTTCAAGAACATGTGT** 595

gi|Oct4pg1|ref|NR_002304.2| **AGACCATCTGCCGCTTTGAGGCTCTGCAGCTTAGCTTCAAGAACATGTGT** 849

.******************** ****************************

gi|Oct4A|ref|NM_002701.4| **AAGCTGCGGCCCTTGCTGCAGAAGTGGGTGGAGGAAGCTGACAACAATGA** 698

gi|Oct4pg3|ref|NR_036440.1| **GAGCTGCGGCCCTTGCTGCAGAAGTGGGTGGAGGAAGCTGACAACAATGA** 643

gi|Oct4pg4|ref|NR_034180.1| **AAGCTGCGGCCCTTGCTGCAGAAGTGGGTGGAGGAAGCTGACAACAATGA** 645

gi|Oct4pg1|ref|NR_002304.2| **AAGCTGCGGCCCTTGCTGCAGAAGTGGGTGGAGGAAGCTGACAACAATGA** 899

.*************************************************

gi|Oct4A|ref|NM_002701.4| **AAATCTTCAGGAGATATGCAAAGCAGAAACCCTCGTGCAGGCCCGAAAGA** 748

gi|Oct4pg3|ref|NR_036440.1| **AAATCTTCAGGAGATATGCAAAGCAGAAACCCTCGTGCAGGCCCGAAAGA** 693

gi|Oct4pg4|ref|NR_034180.1| **AAATCTTCAGGAGACATGCAAAGCAGAAACCCTCTTGCAGGCTCGAAAGA** 695

gi|Oct4pg1|ref|NR_002304.2| **AAATCTTCAGGAGATATGCAAAGCAGAAACCCTCATGCAGGCCCGAAAGA** 949

************** ******************* ******* *******

gi|Oct4A|ref|NM_002701.4| **GAAAGCGAACCAGTATCGAGAACCGAGTGAGAGGCAACC**TGGAGAATTTG 798

gi|Oct4pg3|ref|NR_036440.1| **GAAAGCGAACCAGTATCGAGAACCAAGTGAGAGGCAACC**TGGAGAATTTG 743

gi|Oct4pg4|ref|NR_034180.1| **GAAAGCGAACCAGTATCGAGAACCGAGTGAGAGGCAACC**TGGAGAATTTG 745

gi|Oct4pg1|ref|NR_002304.2| **GAAAGCGAACCAGTATCGAGAACCGAGTGAGAGGCAACC**TGGAGAATTTG 999

************************.*************************

gi|Oct4A|ref|NM_002701.4| TTCCTGCAGTGCCCGAAACCCACACTGCAGCAGATCAGCCACATCGCCCA 848

gi|Oct4pg3|ref|NR_036440.1| TTCCTGCGGTGCCCGAAACCCACACTGCAGCAGATCAGCCACATCGCCCA 793

gi|Oct4pg4|ref|NR_034180.1| TTCCTGCAGTGCCCGAAACCCACACTGCAGCAGATCAGCCACATCGCCCA 795

gi|Oct4pg1|ref|NR_002304.2| TTCCTGCAGTGCCCGAAACCCACACT---GCAGATCAGCCACATCGCCCA 1046

*******.****************** *********************

gi|Oct4A|ref|NM_002701.4| GCAGCTTGGGCTCGAGAAGGATGTGGTCCGAGTGTGGTTCTGTAACCGGC 898

gi|Oct4pg3|ref|NR_036440.1| GCAGCTTGGGCTGGAGAAGGATGTGGTCCGAGTGTGGTTCTGTAACCGGC 843

gi|Oct4pg4|ref|NR_034180.1| GCAGCTTGGGCTCGAGAAGGATGTGGTCCGAGTGTGGTTCTGTAACCGGT 845

gi|Oct4pg1|ref|NR_002304.2| GCAGCTTGGGCTCGAGAAGGATGTGGTCCGAGTGTGGTTCTGTAACCGGC 1096

************ ************************************

gi|Oct4A|ref|NM_002701.4| GCCAGAAGGGCAAGCGATCAAGCAGCGACTATGCACAACGAGAGGATTTT 948

gi|Oct4pg3|ref|NR_036440.1| GCCAGAAGGGCAAGCGATCAAGCAGTGGCTATGCACAACGAGAGGATTTT 893

gi|Oct4pg4|ref|NR_034180.1| GCCAGAAAGGCAAGCAATCAAGCAGCGACTATGCATAACGAGAGGATTTT 895

gi|Oct4pg1|ref|NR_002304.2| GCCAGAAGGGCAAGCGATCAAGCAGCGACTATGCACAACGAGAGGATTTT 1146

*******.*******.********* *.******* **************

gi|Oct4A|ref|NM_002701.4| GAGGCTGCTGGGTCTCCTTTCTCAGGGGGACCAGTGTCCTTTCCTCTGGC 998

gi|Oct4pg3|ref|NR_036440.1| GAGGCTGTTGGGTCTCCTTTCTCAGGGGGACCAGTGTCCTTTCCTCTGGC 943

gi|Oct4pg4|ref|NR_034180.1| GAGGCTGCTGGGTCTCCTTTCTCAGGGGTACCAGTATCCTTTCCTCTGGC 945

gi|Oct4pg1|ref|NR_002304.2| GAGGCTGCTGGGTCTCCTTTCTCAGGGGGACCAGTGTCCTTTCCTCCGGC 1196

******* ******************** ******.********** ***

gi|Oct4A|ref|NM_002701.4| CCCAGGGCCCCATTTTGGTACCCCAGGCTATGGGAGCCCTCACTTCACTG 1048

gi|Oct4pg3|ref|NR_036440.1| CCCAGGGCCCCATTTTGGTACCCCAGGCTATGGGAGCCCTCACTTCACTG 993

gi|Oct4pg4|ref|NR_034180.1| CCCAGGGCCCCATTTTGGTACCCCAGGCTATGGGAGCCCTCACTTCACTG 995

gi|Oct4pg1|ref|NR_002304.2| CCCAGGGCCCCATTTTGGTACCCCAGGCTATGGGAGCCCTCACTTCACTG 1246

**************************************************

gi|Oct4A|ref|NM_002701.4| CACTGTACTCCTCGGTCCCTTTCCCTGAGGGGGAAGCCTTTCCCCCTGTC 1098

gi|Oct4pg3|ref|NR_036440.1| CACTGTACTCCTCGGTCCCTTTCCCTGAGGGGGAAGCCTTTCCCCCTGTC 1043

gi|Oct4pg4|ref|NR_034180.1| CACTGTACTCCTCGGTCCCTTTCCCTGAGGGGGAAGCCTTTCCCCCG-TC 1044

gi|Oct4pg1|ref|NR_002304.2| CACTGTACTCCTCAGTCCCTTTCCCTGAGGGGGAAGTCTTTCCCCCAGTC 1296

*************.********************** ********* **

gi|Oct4A|ref|NM_002701.4| TCCGTCACCACTCTGGGCTCTCCCATGCATTCAAACTGAGGTGCCTGCCC 1148

gi|Oct4pg3|ref|NR_036440.1| TCCGTCACCACTCTGGGCTCTCCCATGCATTCAAACTGAGG--------- 1084

gi|Oct4pg4|ref|NR_034180.1| TCCGTCACCACCCTGGGCTCTCCCATGCATTCAAACTGA----------- 1083

gi|Oct4pg1|ref|NR_002304.2| TCCGTCATCACTCTGGGCTCTCCCATGCATTCAAACTGAGGTGCCTGCCC 1346

******* *** ***************************

gi|Oct4A|ref|NM_002701.4| TTCTAGGAATGGGGGACAGGGGGAGGGGAGGAGCTAGGGAAAGAAAACCT 1198

gi|Oct4pg3|ref|NR_036440.1| --------------------------------------------------

gi|Oct4pg4|ref|NR_034180.1| --------------------------------------------------

gi|Oct4pg1|ref|NR_002304.2| TTCTAGGAATGGGGAACAGGGG-AGGGGAGGAGCTAGGGAAAGAGAACCT 1395

gi|Oct4A|ref|NM_002701.4| GGAGTTTGTGCCAGGGTTTTTGGGATTAAGTTCTTCATTCACTAAGGAAG 1248

gi|Oct4pg3|ref|NR_036440.1| --------------------------------------------------

gi|Oct4pg4|ref|NR_034180.1| --------------------------------------------------

gi|Oct4pg1|ref|NR_002304.2| GGAGTTTGTGGCAGGGCTTTTGGGATTAAGTTCTTCATTCACTAAGGAAG 1445

gi|Oct4A|ref|NM_002701.4| GAATTGGGAACACAAAGGGTGGGGGCAGGGGAGTTTGGGGCAACTGGTTG 1298

gi|Oct4pg3|ref|NR_036440.1| --------------------------------------------------

gi|Oct4pg4|ref|NR_034180.1| --------------------------------------------------

gi|Oct4pg1|ref|NR_002304.2| GAATTGGGAACACTAAGGGTGGGGGCAGGGGAGTTTGGGGCAACTGGTTG 1495

gi|Oct4A|ref|NM_002701.4| GAGGGAAGGTGAAGTTCAATGATGCTCTTGATTTTAATCCCACATCATGT 1348

gi|Oct4pg3|ref|NR_036440.1| --------------------------------------------------

gi|Oct4pg4|ref|NR_034180.1| --------------------------------------------------

gi|Oct4pg1|ref|NR_002304.2| GAGGGAAGGTGAAGTTCAATGATGCTCTTGATTTTAATCCCACATCATGT 1545

gi|Oct4A|ref|NM_002701.4| ATCACTTTTTTCTTAAATAAAGAAGCCTGGGACACAGTAGATAGACACAC 1398

gi|Oct4pg3|ref|NR_036440.1| --------------------------------------------------

gi|Oct4pg4|ref|NR_034180.1| --------------------------------------------------

gi|Oct4pg1|ref|NR_002304.2| ATCACTTTTTTCTTAAATAAAGAAGCCTGGGACACAGTAAAAAAAAAAAA 1595

gi|Oct4A|ref|NM_002701.4| TTAAAAAAAAAAA 1411

gi|Oct4pg3|ref|NR_036440.1| -------------

gi|Oct4pg4|ref|NR_034180.1| -------------

gi|Oct4pg1|ref|NR_002304.2| AAAA--------- 1599

___________________________________________________________________________________________________________

Oct4 primers

F, 5`- GTTGATCCTCGGACCTGGCTA-3`

R, 5`- GGTTGCCTCTCACTCGGTTCT-3` (reverse complement: AGAACCGAGTGAGAGGCAACC)

Amplicon length: 646 bp

Primer sequences are shown in yellow, **bold** is the sequence amplified with these primers.

Purple are polymorphisms in primer annealing sites.

Nucleotide polymorphisms exploited for enzyme specific digestion are marked in red and the whole enzyme recognition site in green.

Restriction sites:

ApaI


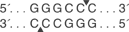


BglI


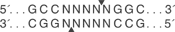


HinfI


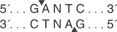


XhoI


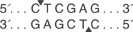

Supplement: Table S2 — CLUSTAL 2.1 multiple sequence alignment of Oct4A, Oct4pg1, Oct4pg3 and Oct4pg4 sequences. Oct4 primers: F, 5′- GTTGATCCTCGGACCTGGCTA-3′; R, 5′- GGTTGCCTCTCACTCGGTTCT-3′; (reverse complement: AGAACCGAGTGAGAGGCAACC). Amplicon length is 646 bp. Primer sequences are shown in yellow, bold is the sequence amplified with these primers. Purple are polymorphisms in primer annealing sites. Nucleotide polymorphisms exploited for enzyme specific digestion are marked in red and the whole enzyme recognition site in green. (DOCX) [file pone.0089546.s003.docx]
