# Supplementary material for: Expression and Differentiation between OCT4A and Its Pseudogenes in Human ESCs and Differentiated Adult Somatic Cells
Source: PLoS One. 2014 Feb 24;9(2):e89546. doi: 10.1371/journal.pone.0089546 (PMC3933561; doi:10.1371/journal.pone.0089546)
Supplement: Table S3 — Alignment of the cloned 646 bp amplicon amplified from embryonic stem cells (H9) – clone 1, 8 and 9– aligned to Oct4A mRNA sequence from GenBank (NM_002701.4). (DOCX) [file pone.0089546.s004.docx]

**Table S3. Alignment of the cloned 646 bp amplicon amplified from embryonic stem cells (H9) – clone 1, 8 and 9 – aligned to Oct4A mRNA sequence from GenBank (NM_002701.4).**

gi|Oct4A|ref|NM_002701.4| GGCACTGCAGGAACAAATTCTCCAGGTTGCCTCTCACTCGGTTCTCGATA 650

gi|Oct4A|646bp ------------------------GGTTGCCTCTCACTCGGTTCTCGATA 26

1_H9_1_646 ------------------------GGTTGCCTCTCACTCGGTTCTCGATA 26

2_H9_8_646 ------------------------GGTTGCCTCTCACTCGGTTCTCGATA 26

3_H9_9_646 ------------------------GGTTGCCTCTCACTCGGTTCTCGATA 26

**************************

gi|Oct4A|ref|NM_002701.4| CTGGTTCGCTTTCTCTTTCGGGCCTGCACGAGGGTTTCTGCTTTGCATAT 700

gi|Oct4A|646bp CTGGTTCGCTTTCTCTTTCGGGCCTGCACGAGGGTTTCTGCTTTGCATAT 76

1_H9_1_646 CTGGTTCGCTTTCTCTTTCGGGCCTGCACGAGGGTTTCTGCTTTGCATAT 76

2_H9_8_646 CTGGTTCGCTTTCTCTTTCGGGCCTGCACGAGGGTTTCTGCTTTGCATAT 76

3_H9_9_646 CTGGTTCGCTTTCTCTTTCGGGCCTGCACGAGGGTTTCTGCTTTGCATAT 76

**************************************************

gi|Oct4A|ref|NM_002701.4| CTCCTGAAGATTTTCATTGTTGTCAGCTTCCTCCACCCACTTCTGCAGCA 750

gi|Oct4A|646bp CTCCTGAAGATTTTCATTGTTGTCAGCTTCCTCCACCCACTTCTGCAGCA 126

1_H9_1_646 CTCCTGAAGATTTTCATTGTTGTCAGCTTCCTCCACCCACTTCTGCAGCA 126

2_H9_8_646 CTCCTGAAGATTTTCATTGTTGTCAGCTTCCTCCACCCACTTCTGCAGCA 126

3_H9_9_646 CTCCTGAAGATTTTCATTGTTGTCAGCTTCCTCCACCCACTTCTGCAGCA 126

**************************************************

gi|Oct4A|ref|NM_002701.4| AGGGCCGCAGCTTACACATGTTCTTGAAGCTAAGCTGCAGAGCCTCAAAG 800

gi|Oct4A|646bp AGGGCCGCAGCTTACACATGTTCTTGAAGCTAAGCTGCAGAGCCTCAAAG 176

1_H9_1_646 AGGGCCGCAGCTTACACATGTTCTTGAAGCTAAGCTGCAGAGCCTCAAAG 176

2_H9_8_646 AGGGCCGCAGCTTACACATGTTCTTGAAGCTAAGCTGCAGAGCCTCAAAG 176

3_H9_9_646 AGGGCCGCAGCTTACACATGTTCTTGAAGCTAAGCTGCAGAGCCTCAAAG 176

**************************************************

gi|Oct4A|ref|NM_002701.4| CGGCAGATGGTCGTTTGGCTGAATACCTTCCCAAATAGAACCCCCAGGGT 850

gi|Oct4A|646bp CGGCAGATGGTCGTTTGGCTGAATACCTTCCCAAATAGAACCCCCAGGGT 226

1_H9_1_646 CGGCAGATGGTCGTTTGGCTGAATACCTTCCCAAATAGAACCCCCAGGGT 226

2_H9_8_646 CGGCAGATGGTCGTTTGGCTGAATACCTTCCCAAATAGAACCCCCAGGGT 226

3_H9_9_646 CGGCAGATGGTCGTTTGGCTGAATACCTTCCCAAATAGAACCCCCAGGGT 226

**************************************************

gi|Oct4A|ref|NM_002701.4| GAGCCCCACATCGGCCTGTGTATATCCCAGGGTGATCCTCTTCTGCTTCA 900

gi|Oct4A|646bp GAGCCCCACATCGGCCTGTGTATATCCCAGGGTGATCCTCTTCTGCTTCA 276

1_H9_1_646 GAGCCCCACATCGGCCTGTGTATATCCCAGGGTGATCCTCTTCTGCTTCA 276

2_H9_8_646 GAGCCCCACATCGGCCTGTGTATATCCCAGGGTGATCCTCTTCTGCTTCA 276

3_H9_9_646 GAGCCCCACATCGGCCTGTGTATATCCCAGGGTGATCCTCTTCTGCTTCA 276

**************************************************

gi|Oct4A|ref|NM_002701.4| GGAGCTTGGCAAATTGCTCGAGTTCTTTCTGCAGAGCTTTGATGTCCTGG 950

gi|Oct4A|646bp GGAGCTTGGCAAATTGCTCGAGTTCTTTCTGCAGAGCTTTGATGTCCTGG 326

1_H9_1_646 GGAGCTTGGCAAATTGCTCGAGTTCTTTCTGCAGAGCTTTGATGTCCTGG 326

2_H9_8_646 GGAGCTTGGCAAATTGCTCGAGTTCTTTCTGCAGAGCTTTGATGTCCTGG 326

3_H9_9_646 GGAGCTTGGCAAATTGCTCGAGTTCTTTCTGCAGAGCTTTGATGTCCTGG 326

**************************************************

gi|Oct4A|ref|NM_002701.4| GACTCCTCCGGGTTTTGCTCCAGCTTCTCCTTCTCCAGCTTCACGGCACC 1000

gi|Oct4A|646bp GACTCCTCCGGGTTTTGCTCCAGCTTCTCCTTCTCCAGCTTCACGGCACC 376

1_H9_1_646 GACTCCTCCGGGTTTTGCTCCAGCTTCTCCTTCTCCAGCTTCACGGCACC 376

2_H9_8_646 GACTCCTCCGGGTTTTGCTCCAGCTTCTCCTTCTCCAGCTTCACGGCACC 376

3_H9_9_646 GACTCCTCCGGGTTTTGCTCCAGCTTCTCCTTCTCCAGCTTCACGGCACC 376

**************************************************

gi|Oct4A|ref|NM_002701.4| AGGGGTGACGGTGCAGGGCTCCGGGGAGGCCCCATCGGAGTTGCTCTCCA 1050

gi|Oct4A|646bp AGGGGTGACGGTGCAGGGCTCCGGGGAGGCCCCATCGGAGTTGCTCTCCA 426

1_H9_1_646 AGGGGTGACGGTGCAGGGCTCCGGGGAGGCCCCATCGGAGTTGCTCTCCA 426

2_H9_8_646 AGGGGTGACGGTGCAGGGCTCCGGGGAGGCCCCATCGGAGTTGCTCTCCA 426

3_H9_9_646 AGGGGTGACGGTGCAGGGCTCCGGGGAGGCCCCATCGGAGTTGCTCTCCA 426

**************************************************

gi|Oct4A|ref|NM_002701.4| CCCCGACTCCTGCTTCGCCCTCAGGCTGAGAGGTCTCCAAGCCGCCTTGG 1100

gi|Oct4A|646bp CCCCGACTCCTGCTTCGCCCTCAGGCTGAGAGGTCTCCAAGCCGCCTTGG 476

1_H9_1_646 CCCCGACTCCTGCTTCGCCCTCAGGCTGAGAGGTCTCCAAGCCGCCTTGG 476

2_H9_8_646 CCCCGACTCCTGCTTCGCCCTCAGGCTGAGAGGTCTCCAAGCCGCCTTGG 476

3_H9_9_646 CCCCGACTCCTGCTTCGCCCTCAGGCTGAGAGGTCTCCAAGCCGCCTTGG 476

**************************************************

gi|Oct4A|ref|NM_002701.4| GGCACTAGCCCCACTCCAACCTGGGGCCCACAGTACGCCATCCCCCCACA 1150

gi|Oct4A|646bp GGCACTAGCCCCACTCCAACCTGGGGCCCACAGTACGCCATCCCCCCACA 526

1_H9_1_646 GGCACTAGCCCCACTCCAACCTGGGGCCCACGGTACGCCATCCCCCCACA 526

2_H9_8_646 GGCACTAGCCCCACTCCAACCTGGGGCCCACAGTACGCCATCCCCCCACA 526

3_H9_9_646 GGCACTAGCCCCACTCCAACCTGGGGCCCACAGTACGCCATCCCCCCACA 526

******************************* ******************

gi|Oct4A|ref|NM_002701.4| GAACTCATACGGCGGGGGGCATGGGGGAATCCCCCACACCTCAGAGCCTG 1200

gi|Oct4A|646bp GAACTCATACGGCGGGGGGCATGGGGGAATCCCCCACACCTCAGAGCCTG 576

1_H9_1_646 GAACTCATACGGCGGGGGGCATGGGGGAATCCCCCACACCTCAGAGCCTG 576

2_H9_8_646 GAACTCATACGGCGGGGGGCATGGGGGAATCCCCCACACCTCAGAGCCTG 576

3_H9_9_646 GAACTCATACGGCGGGGGGCATGGGGGAATCCCCCACACCTCAGAGCCTG 576

**************************************************

gi|Oct4A|ref|NM_002701.4| GCCCAACCCCCGGCCCGATTCCTGGCCCTCCAGGAGGGCCTTGGAAGCTT 1250

gi|Oct4A|646bp GCCCAACCCCCGGCCCGATTCCTGGCCCTCCAGGAGGGCCTTGGAAGCTT 626

1_H9_1_646 GCCCAACCCCCGGCCCGATTCCTGGCCCTCCAGGAGGGCCTTGGAAGCTT 626

2_H9_8_646 GCCCAACCCCCGGCCCGATTCCTGGCCCTCCAGGAGGGCCTTGGAAGCTT 626

3_H9_9_646 GCCCAACCCCCGGCCCGATTCCTGGCCCTCCAGGAGGGCCTTGGGAGCTT 626)

******************************************** *****

gi|Oct4A|ref|NM_002701.4| AGCCAGGTCCGAGGATCAACCCAGCCCGGCTCCGGCCCCCCTGGCCCATC 1300

gi|Oct4A|646bp AGCCAGGTCCGAGGATCAAC------------------------------ 646

1_H9_1_646 AGCCAGGTCCGAGGATCAAC------------------------------ 646

2_H9_8_646 AGCCAGGTCCGAGGATCAAC------------------------------ 646

3_H9_9_646 AGCCAGGTCCGAGGATCAAC------------------------------ 646

********************
