# Supplementary material for: Expression and Differentiation between OCT4A and Its Pseudogenes in Human ESCs and Differentiated Adult Somatic Cells
Source: PLoS One. 2014 Feb 24;9(2):e89546. doi: 10.1371/journal.pone.0089546 (PMC3933561; doi:10.1371/journal.pone.0089546)
Supplement: Table S4 — Alignment of the 646 bp amplicon amplified from fibroblasts grown in 2%O2 and FGF2 (CRL2352 treated) – colony 1, 3, 4, 5, 6 and 7– aligned to Oct4A mRNA sequence from GenBank (NM_002701.4). (DOCX) [file pone.0089546.s005.docx]

**Table S4. Alignment of the 646 bp amplicon amplified from fibroblasts grown in 2%O_2_ and FGF2 (CRL2352 treated) – colony 1, 3, 4, 5, 6 and 7 – aligned to Oct4A mRNA sequence from GenBank (NM_002701.4).**

gi|Oct4A|ref|NM_002701.4| GGCACTGCAGGAACAAATTCTCCAGGTTGCCTCTCACTCGGTTCTCGATA 650

gi|Oct4A|646bp ------------------------GGTTGCCTCTCACTCGGTTCTCGATA 26

5_CRL2352_treated_1 ------------------------GGTTGCCTCTCACTCGGTTCTCGATA 26

7_CRL2352_treated_4 ------------------------GGTTGCCTCTCACTCGGTTCTCGATA 26

10_CRL2352_treated_7 ------------------------GGTTGCCTCTCACTCGGTTCTCGATA 26

33_CRL2352_treated_3 ------------------------GGTTGCCTCTCACTCGGTTCTCGATA 26

35_CRL2352_treated_5 ------------------------GGTTGCCTCTCACTCGGTTCTCGATA 26

36_CRL2352_treated_6 ------------------------GGTTGCCTCTCACTCGGTTCTCGATA 26

**************************

gi|Oct4A|ref|NM_002701.4| CTGGTTCGCTTTCTCTTTCGGGCCTGCACGAGGGTTTCTGCTTTGCATAT 700

gi|Oct4A|646bp CTGGTTCGCTTTCTCTTTCGGGCCTGCACGAGGGTTTCTGCTTTGCATAT 76

5_CRL2352_treated_1 CTGGTTCGCTTTCTCTTTCGGGCCTGCACGAGGGTTTCTGCTTTGCATAT 76

7_CRL2352_treated_4 CTGGTTCGCTTTCTCTTTCGGGCCTGCACGAGGGTTTCTGCTTTGCATAT 76

10_CRL2352_treated_7 CTGGTTCGCTTTCTCTTTCGGGCCTGCACGAGGGTTTCTGCTTTGCATAT 76

33_CRL2352_treated_3 CTGGTTCGCTCTCTCTTTCGGGCCTGCACGAGGGTTTCTGCTTTGCATAT 76

35_CRL2352_treated_5 CTGGTTCGCTTTCTCTTTCGGGCCTGCACGAGGGTTTCTGCTTTGCATAT 76

36_CRL2352_treated_6 CTGGTTCGCTTTCTCTTTCGGGCCTGCACGAGGGTTTCTGCTTTGCATAT 76

********** ***************************************

gi|Oct4A|ref|NM_002701.4| CTCCTGAAGATTTTCATTGTTGTCAGCTTCCTCCACCCACTTCTGCAGCA 750

gi|Oct4A|646bp CTCCTGAAGATTTTCATTGTTGTCAGCTTCCTCCACCCACTTCTGCAGCA 126

5_CRL2352_treated_1 CTCCTGAAGATTTTCATTGTTGTCAGCTTCCTCCACCCACTTCTGCAGCA 126

7_CRL2352_treated_4 CTCCTGAAGATTTTCATTGTTGTCAGCTTCCTCCACCCACTTCTGCAGCA 126

10_CRL2352_treated_7 CTCCTGAAGATTTTCATTGTTGTCAGCTTCCTCCACCCACTTCTGCAGCA 126

33_CRL2352_treated_3 CTCCTGAAGATTTTCATTGTTGTCAGCTTCCTCCACCCACTTCTGCAGCA 126

35_CRL2352_treated_5 CTCCTGAAGATTTTCATTGTTGTCAGCTTCCTCCACCCACTTCTGCAGCA 126

36_CRL2352_treated_6 CTCCTGAAGATTTTCATTGTTGTCAGCTTCCTCCACCCACTTCTGCAGCA 126

**************************************************

gi|Oct4A|ref|NM_002701.4| AGGGCCGCAGCTTACACATGTTCTTGAAGCTAAGCTGCAGAGCCTCAAAG 800

gi|Oct4A|646bp AGGGCCGCAGCTTACACATGTTCTTGAAGCTAAGCTGCAGAGCCTCAAAG 176

5_CRL2352_treated_1 AGGGCCGCAGCTTACACATGTTCTTGAAGCTAAGCTGCAGAGCCTCAAAG 176

7_CRL2352_treated_4 AGGGCCGCAGCTTACACATGTTCTTGAAGCTAAGCTGCAGAGCCTCAAAG 176

10_CRL2352_treated_7 AGGGCCGCAGCTTACACATGTTCTTGAAGCTAAGCTGCAGAGCCTCAAAG 176

33_CRL2352_treated_3 AGGGCCGCAGCTTACACATGTTCTTGAAGCTAAGCTGCAGAGCCTCAAAG 176

35_CRL2352_treated_5 AGGGCCGCAGCTTACACATGTTCTTGAAGCTAAGCTGCAGAGCCTCAAAG 176

36_CRL2352_treated_6 AGGGCCGCAGCTTACACATGTTCTTGAAGCTAAGCTGCAGAGCCTCAAAG 176

**************************************************

gi|Oct4A|ref|NM_002701.4| CGGCAGATGGTCGTTTGGCTGAATACCTTCCCAAATAGAACCCCCAGGGT 850

gi|Oct4A|646bp CGGCAGATGGTCGTTTGGCTGAATACCTTCCCAAATAGAACCCCCAGGGT 226

5_CRL2352_treated_1 CGGCAGATGGTCGTTTGGCTGAATACCTTCCCAAATAGAACCCCCAGGGT 226

7_CRL2352_treated_4 CGGCAGATGGTCGTTTGGCTGAATACCTTCCCAAATAGAACCCCCAGGGT 226

10_CRL2352_treated_7 CGGCAGATGGTCGTTTGGCTGAATACCTTCCCAAATAGAACCCCCAGGGT 226

33_CRL2352_treated_3 CGGCAGATGGTCGTTTGGCTGAATACCTTCCCAAATAGAACCCCCAGGGT 226

35_CRL2352_treated_5 CGGCAGATGGTCGTTTGGCTGAATACCTTCCCAAATAAAACCCCCAGGGT 226

36_CRL2352_treated_6 CGGCAGATGGTCGTTTGGCTGAATACCTTCCCAAATAGAACCCCCAGGGT 226

************************************* ************

gi|Oct4A|ref|NM_002701.4| GAGCCCCACATCGGCCTGTGTATATCCCAGGGTGATCCTCTTCTGCTTCA 900

gi|Oct4A|646bp GAGCCCCACATCGGCCTGTGTATATCCCAGGGTGATCCTCTTCTGCTTCA 276

5_CRL2352_treated_1 GAGCCCCACATCGGCCTGTGTATATCCCAGGGTGATCCTCTTCTGCTTCA 276

7_CRL2352_treated_4 GAGCCCCACATCGGCCTGTGTATATCCCAGGGTGATCCTCTTCTGCTTCA 276

10_CRL2352_treated_7 GAGCCCCACATCGGCCTGTGTATATCCCAGGGTGATCCTCTTCTGCTTCA 276

33_CRL2352_treated_3 GAGCCTCACATCGGCCTGTGTATATCCCAGGGTGATCCTCTTCTGCTTCA 276

35_CRL2352_treated_5 GAGCCCCACATCGGCCTGTGTATATCCCAGGGTGATCCTCTTCTGCTTCA 276

36_CRL2352_treated_6 GAGCCCCACATCGGCCTGTGTATATCCCAGGGTGATCCTCTTCTGCTTCA 276

***** ********************************************

gi|Oct4A|ref|NM_002701.4| GGAGCTTGGCAAATTGCTCGAGTTCTTTCTGCAGAGCTTTGATGTCCTGG 950

gi|Oct4A|646bp GGAGCTTGGCAAATTGCTCGAGTTCTTTCTGCAGAGCTTTGATGTCCTGG 326

5_CRL2352_treated_1 GGAGCTTGGCAAATTGCTCGAGTTCTTTCTGCAGAGCTTTGATGTCCTGG 326

7_CRL2352_treated_4 GGAGCTTGGCAAATTGCTCGAGTTCTTTCTGCAGAGCTTTGATGTCCTGG 326

10_CRL2352_treated_7 GGAGCTTGGCAAATTGCTCGAGTTCTTTCTGCAGAGCTTTGATGTCCTGG 326

33_CRL2352_treated_3 GGAGCTTGGCAAATTGCTCGAGTTCTTTCTGCAGAGCTTTGATGTCCTGG 326

35_CRL2352_treated_5 GGAGCTTGGCAAATTGCTCGAGTTCTTTCTGCAGAGCTTTGATGTCCTGG 326

36_CRL2352_treated_6 GGAGCTTGGCAAATTGCTCGAGTTCTTTCTGCAGAGCTTTGATGTCCTGG 326

**************************************************

gi|Oct4A|ref|NM_002701.4| GACTCCTCCGGGTTTTGCTCCAGCTTCTCCTTCTCCAGCTTCACGGCACC 1000

gi|Oct4A|646bp GACTCCTCCGGGTTTTGCTCCAGCTTCTCCTTCTCCAGCTTCACGGCACC 376

5_CRL2352_treated_1 GACTCCTCCGGGTTTTGCTCCAGCTTCTCCTTCTCCAGCTTCACGGCACC 376

7_CRL2352_treated_4 GACTCCTCCGGGTTTTGCTCCAGCTTCTCCTTCTCCAGCTTCACGGCACC 376

10_CRL2352_treated_7 GACTCCTCCGGGTTTTGCTCCAGCTTCTCCTTCTCCAGCTTCACGGCACC 376

33_CRL2352_treated_3 GACTCCTCCGGGTTTTGCTCCAGCTTCTCCTTCTCCAGCTTCACGGCACC 376

35_CRL2352_treated_5 GACTCCTCCGGGTTTTGCTCCAGCTTCTCCTTCTCCAGCTTCACGGCACC 376

36_CRL2352_treated_6 GACTCCTCCGGGTTTTGCTCCAGCTTCTCCTTCTCCAGCTTCACGGCACC 376

**************************************************

gi|Oct4A|ref|NM_002701.4| AGGGGTGACGGTGCAGGGCTCCGGGGAGGCCCCATCGGAGTTGCTCTCCA 1050

gi|Oct4A|646bp AGGGGTGACGGTGCAGGGCTCCGGGGAGGCCCCATCGGAGTTGCTCTCCA 426

5_CRL2352_treated_1 AGGGGTGACGGTGCAGGGCTCCGGGGAGGCCCCATCGGAGTTGCTCTCCA 426

7_CRL2352_treated_4 AGGGGTGACGGTGCAGGGCTCCGGGGAGGCCCCATCGGAGTTGCTCTCCA 426

10_CRL2352_treated_7 AGGGGTGACGGTGCAGGGCTCCGGGGAGGCCCCATCGGAGTTGCTCTCCA 426

33_CRL2352_treated_3 AGGGGTGACGGTGCAGGGCTCCGGGGAGGCCCCATCGGAGTTGCTCTCCA 426

35_CRL2352_treated_5 AGGGGTGACGGTGCAGGGCTCCGGGGAGGCCCCATCGGAGTTGCTCTCCA 426

36_CRL2352_treated_6 AGGGGTGACGGTGCAGGGCTCCGGGGAGGCCCCATCGGAGTTGCTCTCCA 426

**************************************************

gi|Oct4A|ref|NM_002701.4| CCCCGACTCCTGCTTCGCCCTCAGGCTGAGAGGTCTCCAAGCCGCCTTGG 1100

gi|Oct4A|646bp CCCCGACTCCTGCTTCGCCCTCAGGCTGAGAGGTCTCCAAGCCGCCTTGG 476

5_CRL2352_treated_1 CCCCGACTCCTGCTTCGCCCTCAGGCTGAGAGGTCTCCAAGCCGCCTTGG 476

7_CRL2352_treated_4 CCCCGACTCCTGCTTCGCCCTCAGGCTGAGAGGTCTCCAAGCCGCCTTGG 476

10_CRL2352_treated_7 CCCCGACTCCTGCTTCGCCCTCAGGCTGAGAGGTCTCCAAGCCGCCTTGG 476

33_CRL2352_treated_3 CCCCGACTCCTGCTTCGCCCTCAGGCTGAGAGGTCTCCAAGCCGCCTTGG 476

35_CRL2352_treated_5 CCCCGACTCCTGCTTCGCCCTCAGGCTGAGAGGTCTCCAAGCCGCCTTGG 476

36_CRL2352_treated_6 CCCCGACTCCTGCTTCGCCCTCAGGCTGAGAGGTCTCCAAGCCGCCTTGG 476

**************************************************

gi|Oct4A|ref|NM_002701.4| GGCACTAGCCCCACTCCAACCTGGGGCCCACAGTACGCCATCCCCCCACA 1150

gi|Oct4A|646bp GGCACTAGCCCCACTCCAACCTGGGGCCCACAGTACGCCATCCCCCCACA 526

5_CRL2352_treated_1 GGCACTAGCCCCACTCCAACCTGGGGCCCACAGTACGCCATCCCCCCACA 526

7_CRL2352_treated_4 GGCACTAGCCCCACTCCAACCTGGGGCCCACAGTACGCCATCCCCCCACG 526

10_CRL2352_treated_7 GGCACTAGCCCCACTCCAACCTGGGGCCCACAGTACGCCATCCCCCCACA 526

33_CRL2352_treated_3 GGCACTAGCCCCACTCCAACCTGGGGCCCACAGTACGCCATCCCCCCACA 526

35_CRL2352_treated_5 GGCACTAGCCCCACTCCAACCTGGGGCCCACAGTACGCCATCCCCCCACA 526

36_CRL2352_treated_6 GGCACTAGCCCCACTCCAACCTGGGGCCCACAGTACGCCATCCCCCCACA 526

*************************************************

gi|Oct4A|ref|NM_002701.4| GAACTCATACGGCGGGGGGCATGGGGGAATCCCCCACACCTCAGAGCCTG 1200

gi|Oct4A|646bp GAACTCATACGGCGGGGGGCATGGGGGAATCCCCCACACCTCAGAGCCTG 576

5_CRL2352_treated_1 GAACTCATACGGCGGGGGGCATGGGGGAATCCCCCACGCCTCAGAGCCTG 576

7_CRL2352_treated_4 GAACTCATACGGCGGGGGGCATGGGGGAATCCCCCACACCTCAGAGCCTG 576

10_CRL2352_treated_7 GAACTCATACGGCGGGGGGCATGGGGGAATCCCCCACACCTCAGAGCCTG 576

33_CRL2352_treated_3 GAACTCATACGGCGGGGGGCATGGGGGAATCCCCCACACCTCAGAGCCTG 576

35_CRL2352_treated_5 GAACTCATACGGCGGGGGGCATGGGGGAATCCCCCACACCTCAGAGCCTG 576

36_CRL2352_treated_6 GAACTCATACGGCGGGGGGCATGGGGGAATCCCCCACACCTCAGAGCCTG 576

************************************* ************

gi|Oct4A|ref|NM_002701.4| GCCCAACCCCCGGCCCGATTCCTGGCCCTCCAGGAGGGCCTTGGAAGCTT 1250

gi|Oct4A|646bp GCCCAACCCCCGGCCCGATTCCTGGCCCTCCAGGAGGGCCTTGGAAGCTT 626

5_CRL2352_treated_1 GCCCAACCCCCGGCCCGATTCCTGGCCCTCCAGGAGGGCCTTGGAAGCTT 626

7_CRL2352_treated_4 GCCCAACCCCCGGCCCGATTCCTGGCCCTCCAGGAGGGCCTTGGAAGCTT 626

10_CRL2352_treated_7 GCCCAACCCCCGGCCCGATTCCTGGCCCTCCAGGAGGGCCTTGGAAGCTT 626

33_CRL2352_treated_3 GCCCAACCCCCGGCCCGATTCCTGGCCCTCCAGGAGGGCCTTGGAAGCTT 626

35_CRL2352_treated_5 GCCCAACCCCCGGCCCGATTCCTGGCCCTCCAGGAGGGCCTTGGAAGCTT 626

36_CRL2352_treated_6 GCCCAACCCCCGGCCCGATTCCTGGCCCTCCAGGAGGGCCTTGGAAGCTT 626

**************************************************

gi|Oct4A|ref|NM_002701.4| AGCCAGGTCCGAGGATCAACCCAGCCCGGCTCCGGCCCCCCTGGCCCATC 1300

gi|Oct4A|646bp AGCCAGGTCCGAGGATCAAC------------------------------ 646

5_CRL2352_treated_1 AGCCAGGTCCGAGGATCAAC------------------------------ 646

7_CRL2352_treated_4 AGCCAGGTCCGAGGATCAAC------------------------------ 646

10_CRL2352_treated_7 AGCCAGGTCCGAGGATCAAC------------------------------ 646

33_CRL2352_treated_3 AGCCAGGTCCGAGGATCAAC------------------------------ 646

35_CRL2352_treated_5 AGCCAGGTCCGAGGATCAAC------------------------------ 646

36_CRL2352_treated_6 AGCCAGGTCCGAGGATCAAC------------------------------ 646

********************
