# Supplementary material for: Expression and Differentiation between OCT4A and Its Pseudogenes in Human ESCs and Differentiated Adult Somatic Cells
Source: PLoS One. 2014 Feb 24;9(2):e89546. doi: 10.1371/journal.pone.0089546 (PMC3933561; doi:10.1371/journal.pone.0089546)
Supplement: Table S5 — Alignment of the 646 bp amplicon amplified from control fibroblasts (CRL2352 untreated) – colony 12, 14 and 28– aligned to Oct4pg1 mRNA sequence from GenBank (NR_002304.2). (DOCX) [file pone.0089546.s006.docx]

**Table S5. Alignment of the 646 bp amplicon amplified from control fibroblasts (CRL2352 untreated) – colony 12, 14 and 28 – aligned to Oct4pg1 mRNA sequence from GenBank (NR_002304.2).**

gi|Oct4pg1|ref|NR_002304.2| GGTTGCCTCTCACTCGGTTCTCGATACTGGTTCGCTTTCTCTTTCGGGCC 50

19_CRL2352_untreated_P1_28 GGTTGCCTCTCACTCGGTTCTGGATACTGGTTCGCTTTCTCTTTCGGGCT 50

20_CRL2352_untreated_P1_12 GGTTGCCTCTCACTCGGTTCTGGATACTGGTTCGCTTTCTCTTTCGGGCT 50

21_CRL2352_untreated_P1_14 GGTTGCCTCTCACTCGGTTCTCGATACTGGTTCGCTTTCTCTTTCGGGCC 50

********************* ***************************

gi|Oct4pg1|ref|NR_002304.2| TGCATGAGGGTTTCTGCTTTGCATATCTCCTGAAGATTTTCATTGTTGTC 100

19_CRL2352_untreated_P1_28 TGCATGAGGGTTTCTGCTTTGCATATCTCCTGAAGATTTTCATTGTTGTC 100

20_CRL2352_untreated_P1_12 TGCATGAGGGTTTCTGCTTTGCATATCTCCTGAAGATTTTCATTGTTGTC 100

21_CRL2352_untreated_P1_14 TGCATGAGGGTTTCTGCTTTGCATATCTCCTGAAGATTTTCATTGTTGTC 100

**************************************************

gi|Oct4pg1|ref|NR_002304.2| AGCTTCCTCCACCCACTTCTGCAGCAAGGGCCGCAGCTTACACATGTTCT 150

19_CRL2352_untreated_P1_28 AGCTTCCTCCACCCACTTCTGCAGCAAGGGCCGCAGCTTACACATGTTCT 150

20_CRL2352_untreated_P1_12 AGCTTCCTCCACCCACTTCTGCAGCAAGGGCCGCAGCTTACACATGTTCT 150

21_CRL2352_untreated_P1_14 AGCTTCCTCCACCCACTTCTGCAGCAAGGGCCGCAGCTTGCACATGTTCT 150

*************************************** **********

gi|Oct4pg1|ref|NR_002304.2| TGAAGCTAAGCTGCAGAGCCTCAAAGCGGCAGATGGTCTTTTGGCTGAAC 200

19_CRL2352_untreated_P1_28 TGAAGCTAAGCTGCAGAGCCTCAAAGCGGCAGATGGTCTTTTGGCTGAAC 200

20_CRL2352_untreated_P1_12 TGAAGCTAAGCTGCAGAGCCTCAAAGCGGCAGATGGTCTTTTGGCTGAAC 200

21_CRL2352_untreated_P1_14 TGAAGCTAAGCTGCAGAGCCTCAAAGCGGCAGATGGTCTTTTGGCTGAAC 200

**************************************************

gi|Oct4pg1|ref|NR_002304.2| ACCTTCCCAAATAGAACCCCCAGGATGAGCCCCACATCGGCCTGTGTATA 250

19_CRL2352_untreated_P1_28 ACCTTCTCAAATAGAACCCCCAGGATGAGCCCCACATCGGCCTGTGTATA 250

20_CRL2352_untreated_P1_12 ACCTTCTCAAATAGAACCCCCAGGATGAGCCCCACATCGGCCTGTGTATA 250

21_CRL2352_untreated_P1_14 ACCTTCCCAAATAGAACCCCCAGGATGAGCCCCACATCGGCCTGTGTATA 250

****** *******************************************

gi|Oct4pg1|ref|NR_002304.2| TCCCAGGGTGATCCTCTTCTGCTTCAGGAGCTTGGCAAATTGCTCGAGTT 300

19_CRL2352_untreated_P1_28 TCCCAGGGTGATCCTCTTCTGCTTCAGGAGCTTGGCAAATTGCTCGAGTT 300

20_CRL2352_untreated_P1_12 TCCCAGGGTGATCCTCTTCTGCTTCAGGAGCTTGGCAAATTGCTCGAGTT 300

21_CRL2352_untreated_P1_14 TCCCAGGGTGATCCTCTTCTGCTTCAGGAGCTTGGCAAATTGCTCGAGTT 300

**************************************************

gi|Oct4pg1|ref|NR_002304.2| CTTTCTGCAGAGCTTTGATGTCCTGGGACTTCTCCGGGTTTTGCTCTAGC 350

19_CRL2352_untreated_P1_28 CTTTCTGCAGAGCTTTGATGTCCTGGGACTTCTCCGGGTTTTGCTCTAGC 350

20_CRL2352_untreated_P1_12 CTTTCTGCAGAGCTTTGATGTCCTGGGACTTCTCCGGGTTTTGCTCTAGC 350

21_CRL2352_untreated_P1_14 CTTTCTGCAGAGTTTTGATGTCCTGGGACTTCTCCGGGTTTTGCTCTAGC 350

************ *************************************

gi|Oct4pg1|ref|NR_002304.2| TTCTCCTTCTCCAGCTTCACGGCACCAGGGGGGACGGTGCAGGGTTCCGG 400

19_CRL2352_untreated_P1_28 TTCTCCTTCTCCAGCTTCACGGCACCAGGGGGGACGGTGCAGGGTTCCGG 400

20_CRL2352_untreated_P1_12 TTCTCCTTCTCCAGCTTCACGGCACCAGGGGGGACGGTGCAGGGTTCCGG 400

21_CRL2352_untreated_P1_14 TTCTCCTTCTCCAGCTTCACGGCACCAGGGGGGACGGTGCAGGGTTCCGG 400

**************************************************

gi|Oct4pg1|ref|NR_002304.2| GGAGGCCCCATTGGAGTTGCTCTCCACCCCGACTCCTGCTTCGCTCTCAG 450

19_CRL2352_untreated_P1_28 GGAGGCCCCATTGGAGTTGCTCTCCACCCCGACTCCTGCTTCGCTCTCAG 450

20_CRL2352_untreated_P1_12 GGAGGCCCCATTGGAGTTGCTCTCCACCCCGACTCCTGCTTCGCTCTCAG 450

21_CRL2352_untreated_P1_14 GGAGGCCCCATTGGAGTTGCTCTCCACCCCGACTCCTGCTTCGCTCTCAG 450

**************************************************

gi|Oct4pg1|ref|NR_002304.2| GCTGAGAGGTCTCCAAGCCGCCTTGGGGCACTAGCCCCACTCCAACCTGA 500

19_CRL2352_untreated_P1_28 GCTGAGAGGTCTCCAAGCCGCCTTGGGGCACTAGCCCCACTCCAACCTGA 500

20_CRL2352_untreated_P1_12 GCTGAGAGGTCTCCAAGCCGCCTTGGGGCACTAGCCCCACTCCAACCTGA 500

21_CRL2352_untreated_P1_14 GCTGAGAGGTCTCCAAGCCGCCTTGGGGCACTAGCCCCACTCCAACCTGA 500

**************************************************

gi|Oct4pg1|ref|NR_002304.2| GGCCCACAGTACGCCATCCCCCCACATAACTCATACGGCGGGGGGCAAGG 550

19_CRL2352_untreated_P1_28 GGCCCACAGTACGCCATCCCCCCACATAACTCATACGGCGGGGGGCAAGG 550

20_CRL2352_untreated_P1_12 GGCCCACAGTACGCCATCCCCCCACATAACTCATACGGCGGGGGGCAAGG 550

21_CRL2352_untreated_P1_14 GGCCCACAGTACGCCATCCCCCCACATAACTCATACGGCGGGGGGCAAGG 550

**************************************************

gi|Oct4pg1|ref|NR_002304.2| GGGAATCCCCCACACCTCAGAGCCTGGCCCAACCCCCGGCCCGATTCCTG 600

19_CRL2352_untreated_P1_28 GGGAATCCCCCACACCTCAGAGCCTGGCCCAACCCCCGGCCCGATTCCTG 600

20_CRL2352_untreated_P1_12 GGGAATCCCCCACACCTCAGAGCCTGGCCCAACCCCCGGCCCGATTCCTG 600

21_CRL2352_untreated_P1_14 GGGAATCCCCCACACCTCAGAGCCTGGCCCAACCCCCGGCCCGATTCCTG 600

**************************************************

gi|Oct4pg1|ref|NR_002304.2| GCCCTCCAGGAGGGCCTTGGAAGCTTAGCCAGGTCAGAGGATCAAC- 646

19_CRL2352_untreated_P1_28 GCCCTCCAGGAGGGCCTTGGAAGCTTAGCCAGGTCCGAGGATCAAC- 646

20_CRL2352_untreated_P1_12 GCCCTCCAGGAGGGCCTTGGAAGCTTAGCCAGGTCCGAGGATCAAC- 646

21_CRL2352_untreated_P1_14 GCCCTCCAGGAGGGCCTTGGAAGCTTAGCCAGGTCCGAGGAT-AACA 646

*********************************** ****** ***
