# Supplementary material for: Expression and Differentiation between OCT4A and Its Pseudogenes in Human ESCs and Differentiated Adult Somatic Cells
Source: PLoS One. 2014 Feb 24;9(2):e89546. doi: 10.1371/journal.pone.0089546 (PMC3933561; doi:10.1371/journal.pone.0089546)
Supplement: Table S6 — Alignment of the 646 bp amplicon amplified from control fibroblasts (CRL2352 untreated) – colony 3, 7 and 11– aligned to Oct4pg3 mRNA sequence from GenBank (NR_036440.1). (DOCX) [file pone.0089546.s007.docx]

**Table S6. Alignment of the 646 bp amplicon amplified from control fibroblasts (CRL2352 untreated) – colony 3, 7 and 11 – aligned to Oct4pg3 mRNA sequence from GenBank (NR_036440.1).**

gi|Oct4pg3|ref|NR_036440.1| GGTTGCCTCTCACTTG-GTTCTCGATACTGGTTCGCTTTCTCTTTCGGGC 49

22_CRL2352_untreated_P3_11 GGTTGCCTCTCACTCG-GTTCTCGATACTGGTTCGCTTTCTCTTTCCGGC 49

23_CRL2352_untreated_P3_3 GGTTGCCTCTCACTCG-GTTCTCGATACTGGTTCGCTTTCTCTTTCCGGC 49

24_CRL2352_untreated_P3_7 GGTTGCCTCTCACTCG-GTTCTCGATACTGGTTCGCTTTCTCTTTCCGGC 49

49_CRL2352_untreated_P3_11 GGTTGCCTCTCACTCGAGTTCTCGATACTGGTTCGCTTTCTCTTTCCGGC 50

50_CRL2352_untreated_P3_3 GGTTGCCTCTCACTCG-GTTCTCGATACTGGTTCGCTTTCTCTTTCCGGC 49

51_CRL2352_untreated_P3_7 GGTTGCCTCTCACTCG-GTTCTCGATACTGGTTCGCTTTCTCTTTCCGGC 49

************** * ***************************** ***

gi|Oct4pg3|ref|NR_036440.1| CTGCACGAGGGTTTCTGCT-TTGCATATCTCCTGAAGATTTTCATTGTTG 98

22_CRL2352_untreated_P3_11 CTGCACGAGGGTTTCTGCT-TTGCATATCTCCTGAAGATTTTCATTGTTG 98

23_CRL2352_untreated_P3_3 CTGCACGAGGGTTTCTGCT-TTGCATATCTCCTGAAGATTTTCATTGTTG 98

24_CRL2352_untreated_P3_7 CTGCACGAGGGTTTCTGCT-TTGCATATCTCCTGAAGATTTTCATTGTTG 98

49_CRL2352_untreated_P3_11 CTGCACGAGGGTTTCTGCT-TTGCATATCTCCTGAAGATTTTCATTGTTG 99

50_CRL2352_untreated_P3_3 CTGCACGAGGGTTTCTGCT-TTGCATATCTCCTGAAGATTTTCATTGTTG 98

51_CRL2352_untreated_P3_7 CTGCACGAGGGTTTCTGCTGTTGCATATCTCCTGAAGATTTTCATTGTTG 99

******************* ******************************

gi|Oct4pg3|ref|NR_036440.1| TCAGCTTCCTCCACCCACTTCTGCAGCAAGGGCCGCAGCTCACACATGTT 148

22_CRL2352_untreated_P3_11 TCAGCTTCCTCCACCCACTTCTGCAGCAAGGGCCGCAGCTCACACATGTT 148

23_CRL2352_untreated_P3_3 TCAGCTTCCTCCACCCACCTCTGCAGCAAGGGCCGCAGCTCACACATGTT 148

24_CRL2352_untreated_P3_7 TCAGCTTCCTCCACCCACTTCTGCAGCAAGGGCCGCAGCTCACACATGTT 148

49_CRL2352_untreated_P3_11 TCAGCTTCCTCCACCCACTTCTGCAGCAAGGGCCGCAGCTCACACATGTT 149

50_CRL2352_untreated_P3_3 TCAGCTTCCTCCACCCACCTCTGCAGCAAGGGCCGCAGCTCACACATGTT 148

51_CRL2352_untreated_P3_7 TCAGCTTCCTCCACCCACTTCTGCAGCAAGGGCCGCAGCTCACACATGTT 149

****************** *******************************

gi|Oct4pg3|ref|NR_036440.1| CTTGAAGCTAAGCTGCAGAGCCTCAAAGCGGCAGATGGTCGTTTGGCTGA 198

22_CRL2352_untreated_P3_11 CTTGAAGCTAAGCTGCAGAGCCTCAAAGCGGCAGATGGTCGTTTGGCTGA 198

23_CRL2352_untreated_P3_3 CTTGAAGCTAAGCTGCAGAGCCTCAAAGCGGCAGATGGTCGTTTGGCTGA 198

24_CRL2352_untreated_P3_7 CTTGAAGCTAAGCTGCAGAGCCTCAAAGCGGCAGATGGTCGTTTGGCTGA 198

49_CRL2352_untreated_P3_11 CTTGAAGCTAAGCTGCAGAGCCTCAAAGCGGCAGATGGTCGTTTGGCTGA 199

50_CRL2352_untreated_P3_3 CTTGAAGCTAAGCTGCAGAGCCTCAAAGCGGCAGATGGTCGTTTGGCTGA 198

51_CRL2352_untreated_P3_7 CTTGAAGCTAAGCTGCAGAGCCTCAAAGCGGCAGATGGTCGTTTGGCTGA 199

**************************************************

gi|Oct4pg3|ref|NR_036440.1| ACACCTTCCCAAATAGAACCCCCAGGGTGAGCCACATCGGCCTGTGTATA 248

22_CRL2352_untreated_P3_11 ACACCTTCCCAAATAGAACCCCCAGGGTGAGCCACATCGGCCTGTGTATA 248

23_CRL2352_untreated_P3_3 ACACCCTCCCAAATAGAACCCCCAGGGTGAGCCACATCGGCCTGTGTATA 248

24_CRL2352_untreated_P3_7 ACACCTTCCCAAATAGAACCCCCAGGGTGAGCCACATCGGCCTGTGTATA 248

49_CRL2352_untreated_P3_11 ACACCTTCCCAAATAGAACCCCCAGGGTGAGCCACATCGGCCTGTGTATA 249

50_CRL2352_untreated_P3_3 ACACCCTCCCAAATAGAACCCCCAGGGTGAGCCACATCGGCCTGTGTATA 248

51_CRL2352_untreated_P3_7 ACACCTTCCCAAATAGAACCCCCAGGGTGAGCCACATCGGCCTGTGTATA 249

***** ********************************************

gi|Oct4pg3|ref|NR_036440.1| TCCCAGGGTGATCCTCTTCTGCTTCAGGAGCTTGGCAAATTGCTCGAGTT 298

22_CRL2352_untreated_P3_11 TCCCAGGGTGATCCTCTTCTGCTTCAGGAGCTTGGCAAATTGCTCGAGTT 298

23_CRL2352_untreated_P3_3 TCCCAGGGTGATCCTCTTCTGCTTCAGGAGCTTGGCAAATTGCTCGAGTT 298

24_CRL2352_untreated_P3_7 TCCCAGGGTGATCCTCTTCTGCTTCAGGAGCTTGGCAAATTGCTCGAGTT 298

49_CRL2352_untreated_P3_11 TCCCAGGGTGATCCTCTTCTGCTTCAGGAGCTTGGCAAATTGCTCGAGTT 299

50_CRL2352_untreated_P3_3 TCCCAGGGTGATCCTCTTCTGCTTCAGGAGCTTGGCAAATTGCTCGAGTT 298

51_CRL2352_untreated_P3_7 TCCCAGGGTGATCCTCTTCTGCTTCAGGAGCTTGGCAAATTGCTCGAGTT 299

**************************************************

gi|Oct4pg3|ref|NR_036440.1| CTTTCTGCAGAGCTTTGATGTCCTGGGACTCCTCCGGGTTTTGCTCCAGC 348

22_CRL2352_untreated_P3_11 CTTTCTGCAGAGCTTTGATGTCCTGGGACTCCTTCGGGTTTTGCTCCAGC 348

23_CRL2352_untreated_P3_3 CTTTCTGCAGAGCTTTGATGTCCTGGGACTCCTTCGGGTTTTGCTCCAGC 348

24_CRL2352_untreated_P3_7 CTTTCTGCAGAGCTTTGATGTCCTGGGACTCCTTCGGGTTTTGCTCCAGC 348

49_CRL2352_untreated_P3_11 CTTTCTGCAGAGCTTTGATGTCCTGGGACTCCTTCGGGTTTTGCTCCAGC 349

50_CRL2352_untreated_P3_3 CTTTCTGCAGAGCTTTGATGTCCTGGGACTCCTTCGGGTTTTGCTCCAGC 348

51_CRL2352_untreated_P3_7 CTTTCTGCAGAGCTTTGATGTCCTGGGACTCCTTCGGGTTTTGCTCCAGC 349

********************************* ****************

gi|Oct4pg3|ref|NR_036440.1| TTCTCCTTCTCCAGCTTCACGGCACCAGAGGGGACGGTGCAGGGCTCCGG 398

22_CRL2352_untreated_P3_11 TTCTCCTTCTCCAGCTTCACGGCACCAGAGGGGACGGTGCAGGGCTCCGG 398

23_CRL2352_untreated_P3_3 TTCTCCTTCTCCAGCTTCACGGCACCAGAGGGGACGGTGCAGGGCTCCGG 398

24_CRL2352_untreated_P3_7 TTCTCCTTCTCCAGCTTCACGGCACCAGAGGGGACGGTGCAGGGCTCCGG 398

49_CRL2352_untreated_P3_11 TTCTCCTTCTCCAGCTTCACGGCACCAGAGGGGACGGTGCAGGGCTCCGG 399

50_CRL2352_untreated_P3_3 TTCTCCTTCTCCAGCTTCACGGCACCAGAGGGGACGGTGCAGGGCTCCGG 398

51_CRL2352_untreated_P3_7 TTCTCCTTCTCCAGCTTCACGGCACCAGAGGGGACGGTGCAGGGCTCCGG 399

**************************************************

gi|Oct4pg3|ref|NR_036440.1| GGAGGCCCCATCGGAGTTGCTCTCCACCCCGACTCCTGCTTCGCCCTCAG 448

22_CRL2352_untreated_P3_11 GGAGGCCCCATCGGAGTTGCTCTCCACCCCGACTCCTGCTTCGCCCTCAG 448

23_CRL2352_untreated_P3_3 GGAGGCCCCATCGGAGTTGCTCTCCACCCCGACTCCTGCTTCGCCCTCAG 448

24_CRL2352_untreated_P3_7 GGAGGCCCCATCGGAGTTGCTCTCCACCCCGACTCCTGCTTCGCCCTCAG 448

49_CRL2352_untreated_P3_11 GGAGGCCCCATCGGAGTTGCTCTCCACCCCGACTCCTGCTTCGCCCTCAG 449

50_CRL2352_untreated_P3_3 GGAGGCCCCATCGGAGTTGCTCTCCACCCCGACTCCTGCTTCGCCCTCAG 448

51_CRL2352_untreated_P3_7 GGAGGCCCCATCGGAGTTGCTCTCCACCCCGACTCCTGCTTCGCCCTCAG 449

**************************************************

gi|Oct4pg3|ref|NR_036440.1| GCTGAGAGGTCTCCAAGCCGTCTTGGGGCACTAGCCCCACTCCAGTCTGA 498

22_CRL2352_untreated_P3_11 GCTGAGAGGTCTCCAAGCCGTCTTGGGGCACTAGCCCCACTCCAGTCTGA 498

23_CRL2352_untreated_P3_3 GCTGAGAGGTCTCCAAGCCGTCTTGGGGCACTAGCCCCACTCCAGTCTGA 498

24_CRL2352_untreated_P3_7 GCTGAGAGGTCTCCAAGCCGTCTTGGGGCACTAGCCCCACTCCAGTCTGA 498

49_CRL2352_untreated_P3_11 GCTGAGAGGTCTCCAAGCCGTCTTGGGGCACTAGCCCCACTCCAGTCTGA 499

50_CRL2352_untreated_P3_3 GCTGAGAGGTCTCCAAGCCGTCTTGGGGCACTAGCCCCACTCCAGTCTGA 498

51_CRL2352_untreated_P3_7 GCTGAGAGGTCTCCAAGCCGTCTTGGGGCACTAGCCCCACTCCAGTCTGA 499

**************************************************

gi|Oct4pg3|ref|NR_036440.1| GGCCCACAGTACGCCATCCCCCCGCAGAACTCATACGGCGGGGGACATGG 548

22_CRL2352_untreated_P3_11 GGCCCACAGTACGCCATCCCCCCGCAGAACTCATACGGCGGGGGACATGG 548

23_CRL2352_untreated_P3_3 GGCCCACAGTACGCCATCCCCCCGCAGAACTCATACGGCGGGGGACATGG 548

24_CRL2352_untreated_P3_7 GGCCCACAGTACGCCATCCCCCCGCAGAGCTCATACGGCGGGGGACATGG 548

49_CRL2352_untreated_P3_11 GGCCCACAGTACGCCATCCCCCCGCAGAACTCATACGGCGGGGGACATGG 549

50_CRL2352_untreated_P3_3 GGCCCACAGTACGCCATCCCCCCGCAGAACTCATACGGCGGGGGACATGG 548

51_CRL2352_untreated_P3_7 GGCCCACAGTACGCCATCCCCCCGCAGAGCTCATACGGCGGGGGACATGG 549

**************************** *********************

gi|Oct4pg3|ref|NR_036440.1| GGGAATCCCCCACTCCTCAGAGCCTGGCCCAAACCCCGGCCCGATTCCTG 598

22_CRL2352_untreated_P3_11 GGGAATCCCCCACTCCTCAGAGCCTGGCCCAAACCCCGGCCCGATTCCTG 598

23_CRL2352_untreated_P3_3 GGGAATCCCCCACTCCTCAGAGCGTGACCCAAACCCCGGCCCGATTCCTG 598

24_CRL2352_untreated_P3_7 GGGAATCCCCCACTCCTCAGAGCCTGGCCCAAACCCCGGCCCGATTCCTG 598

49_CRL2352_untreated_P3_11 GGGAATCCCCCACTCCTCAGAGCCTGGCCCAAACCCCGGCCCGATTCCTG 599

50_CRL2352_untreated_P3_3 GGGAATCCCCCACTCCTCAGAGCCTGGCCCAAACCCCGGCCCGATTCCTG 598

51_CRL2352_untreated_P3_7 GGGAATCCCCCACTCCTCAGAGCCTGGCCCAAACCCCGGCCCGATTCCTG 599

*********************** ** ***********************

gi|Oct4pg3|ref|NR_036440.1| GCCCTCCAGGAGGGCCTTGGAAGCTTAGCCAGGTCCGAGGATCAAC 644

22_CRL2352_untreated_P3_11 GCCCTCCAGGAGGGCCTTGGAAGCTTAGCCAGGTCCGAGGATCAAC 644

23_CRL2352_untreated_P3_3 GCCCTCCAGGAGGGCCTTGGAAGCTTAGCCAGGTCCGAGGATCAAC 644

24_CRL2352_untreated_P3_7 GCCCTCCAGGAGGGCCTTGGAAGCTTAGCCAGGTCCGAGGATCAAC 644

49_CRL2352_untreated_P3_11 GCCCTCCAGGAGGGCCTTGGAAGCTTAGCCAGGTCCGAGGATCAAC 645

50_CRL2352_untreated_P3_3 GCCCTCCAGGAGGGCCTTGGAAGCTTAGCCAGGTCCGAGGATCAAC 644

51_CRL2352_untreated_P3_7 GCCCTCCAGGAGGGCCTTGGAAGCTTAGCCAGGTCCGAGGATCAAC 645

**********************************************
