# Supplementary material for: Expression and Differentiation between OCT4A and Its Pseudogenes in Human ESCs and Differentiated Adult Somatic Cells
Source: PLoS One. 2014 Feb 24;9(2):e89546. doi: 10.1371/journal.pone.0089546 (PMC3933561; doi:10.1371/journal.pone.0089546)
Supplement: Table S7 — Alignment of the 646 bp amplicon amplified from control fibroblasts (CRL2352 untreated) – colony 2 and 5– aligned to Oct4pg4 mRNA sequence from GenBank (NR_034180.1). (DOCX) [file pone.0089546.s008.docx]

**Table S7. Alignment of the 646 bp amplicon amplified from control fibroblasts (CRL2352 untreated) – colony 2 and 5 – aligned to Oct4pg4 mRNA sequence from GenBank (NR_034180.1).**

gi|Oct4pg4|ref|NR_034180.1| GGTTGCCTCTCACTCG-GTTCTCGATACT-GGTTCGCTTTCTCTTTCGAG 48

25_CRL2352_untreated_P4_2 GGTTGCCTCTCACTCGCGTTCTCGATACTCGGTTCGCTTTCTCTTTCGAG 50

27_CRL_2352_untreated_P4_5 GGTTGCCTCTCACTCG-GTTCTCGATACT-GGTTCGCTTTCTCTTTCGAG 48

**************** ************ ********************

gi|Oct4pg4|ref|NR_034180.1| CCTGCAAGAGGGTTTCTGCTTTGCATGTCTCCTGAAGATTTTCATTGTTG 98

25_CRL2352_untreated_P4_2 CCTGCAAGAGGGTTTCTGCTTTGCATGTCTCCTGAAGATTTTCATTGTTG 100

27_CRL_2352_untreated_P4_5 CCTGCAAGAGGGTTTCTGCTTTGCATGTCTCCTGAAGATTTTCATTGTTG 98

**************************************************

gi|Oct4pg4|ref|NR_034180.1| TCAGCTTCCTCCACCCACTTCTGCAGCAAGGGCCGCAGCTTACACATGTT 148

25_CRL2352_untreated_P4_2 TCAGCTTCCTCCACCCACTTCTGCAGCAAGGGCCGCAGCTTACACACGTT 150

27_CRL_2352_untreated_P4_5 TCAGCTTCCTCCACCCACTTCTGCAGCAAGGGCCGCAGCTTACACATGTT 148

********************************************** ***

gi|Oct4pg4|ref|NR_034180.1| CTTGAAGCTAAGCTGCAGACCCTCAAAGCGGCAGATGGTCGTTTGGCTGA 198

25_CRL2352_untreated_P4_2 CTTGAAGCTAAGCTGCAGACCCTCAAAGCGGCAGATGGTCGTTTGGCTGA 200

27_CRL_2352_untreated_P4_5 CTTGAAGCTAAGCTGCAGACCCTCAAAGCGGCAGATGGTCGTTTGGCTGA 198

**************************************************

gi|Oct4pg4|ref|NR_034180.1| ACACCTTCCCAAATAGAACCCCCAGGGTGAGCCCCACATCGGCCTGTGTA 248

25_CRL2352_untreated_P4_2 ACACCTTCCCAAATAGAACCCCCAGGGTGAGCCCCACATCGGCCTGTGTA 250

27_CRL_2352_untreated_P4_5 ACACCTTCCCAAATAGAACCCCCAGGGTGAGCCCCACATCGGCCTGTGTA 248

**************************************************

gi|Oct4pg4|ref|NR_034180.1| TATCCCAGGGTGATCCTCTTCTGCTTCAGGAGCTTGGCAAATTGTTCGAG 298

25_CRL2352_untreated_P4_2 TATCCCAGGGTGATCCTCTTCTGCTTCAGGAGCTTGGCAAATTGTTCGAG 300

27_CRL_2352_untreated_P4_5 TATCCCAGGGTGATCCTCTTCTGCTTCAGGAGCTTGGCAAATTGTTCGAG 298

**************************************************

gi|Oct4pg4|ref|NR_034180.1| TTCTTTCTGCAGAGCTTTGATGTTCTGGGACTCCTGCGGGTTTTGCTCCA 348

25_CRL2352_untreated_P4_2 TTCTTTCTGCAGAGCTTTGATGTTCTGGGACTCCTGCGGGTTTTGCTCCA 350

27_CRL_2352_untreated_P4_5 TTCTTTCTGCAGAGCTTTGATGTTCTGGGACTCCTGCGGGTTTTGCTCCA 348

**************************************************

gi|Oct4pg4|ref|NR_034180.1| GCTTCTCCTTCTCCAGTTTCACGGCACCAGGGGGGACGGTGCAGGGCTCC 398

25_CRL2352_untreated_P4_2 GCTTCTCCTTCTCCAGTTTCACGGCACCAGGGGGGACGGTGCAGGGCTCC 400

27_CRL_2352_untreated_P4_5 GCTTCTCCTTCTCCAGTTTCACGGCACCAGGGGGGACGGTGCAGGGCTCC 398

**************************************************

gi|Oct4pg4|ref|NR_034180.1| AGGGAGGTGCCATCGGAGTTGCTCTCCACCCTGACTCCTGCTTCGCCCTC 448

25_CRL2352_untreated_P4_2 AGGGAGGTGCCATCGGAGTTGCTCTCCACCCTGACTCCTGCTTCGCCCTC 450

27_CRL_2352_untreated_P4_5 AGGGAGGTGCCATCGGAGTTGCTCTCCACCCTGACTCCTGCTTCGCCCTC 448

**************************************************

gi|Oct4pg4|ref|NR_034180.1| AGGCTGAGAGGTCTCCAAGCCGCCTTGGGGCACTAGCCGCACTCCAACCT 498

25_CRL2352_untreated_P4_2 AGGCTGAGAGGTCTCCAAGCCGCCTTGGGGCACTAGCCGCACTCCAACCT 500

27_CRL_2352_untreated_P4_5 AGGCTGAGAGGTCTCCAAGCCGCCTTGGGGCACTAGCCGCACTCCAACCT 498

**************************************************

gi|Oct4pg4|ref|NR_034180.1| GAGGCCCACAGTACGCCATCCCCCCACAGAACTCATACAGCGGGGGGCAT 548

25_CRL2352_untreated_P4_2 GAGGCCCACAGTACGCCATCCCCCCACAGAACTCATACAGCGGGGGGCAT 550

27_CRL_2352_untreated_P4_5 GAGGCCCACAGTACGCCATCCCCCCACAGAACTCATACAGCGGGGGGCAT 548

**************************************************

gi|Oct4pg4|ref|NR_034180.1| GGGGGAATCCCCCACACCTCAGAGCCTGACCCAACTCCCGGCCCGATTCC 598

25_CRL2352_untreated_P4_2 GGGGGAATCCCCCACACCTCAGAGCCTGACCCAACTCCCGGCCCGATTCC 600

27_CRL_2352_untreated_P4_5 GGGGGAATCCCCCACACCTCAGAGCCTGACCCAACTCCCGGCCCGATTCC 598

**************************************************

gi|Oct4pg4|ref|NR_034180.1| TGGCCCTCCAGGAGGGCCTTGGAAGCTTAGCCAGGTCCGAGGATCAAC 646

25_CRL2352_untreated_P4_2 TGGCCCTCCAGGAGGGCCTTGGAAGCTTAGCCAGGTCCGAGGATCAAC 648

27_CRL_2352_untreated_P4_5 TGGCCCTCCAGGAGGGCCTTGGAAGCTTAGCCAGGTCCGAGGATCAAC 646

************************************************
